# The COMET toolkit for composing customizable genetic programs in mammalian cells

**DOI:** 10.1038/s41467-019-14147-5

**Published:** 2020-02-07

**Authors:** Patrick S. Donahue, Joseph W. Draut, Joseph J. Muldoon, Hailey I. Edelstein, Neda Bagheri, Joshua N. Leonard

**Affiliations:** 1 Department of Chemical and Biological Engineering, Northwestern University, Evanston, IL 60208 USA; 2 Interdisciplinary Biological Sciences Program, Northwestern University, Evanston, IL 60208 USA; 3 Medical Scientist Training Program, Northwestern University Feinberg School of Medicine, Chicago, IL 60611 USA; 4 Center for Synthetic Biology, Northwestern University, Evanston, IL 60208 USA; 5 Chemistry of Life Processes Institute, Northwestern University, Evanston, IL 60208 USA; 6 Member, Robert H. Lurie Comprehensive Cancer Center, Northwestern University, Evanston, IL 60208 USA; 7 Biology and Chemical Engineering, University of Washington, Seattle, WA 98195 USA

**Keywords:** Synthetic biology, Gene expression, Transcriptional regulatory elements

## Abstract

Engineering mammalian cells to carry out sophisticated and customizable genetic programs requires a toolkit of multiple orthogonal and well-characterized transcription factors (TFs). To address this need, we develop the COmposable Mammalian Elements of Transcription (COMET)—an ensemble of TFs and promoters that enable the design and tuning of gene expression to an extent not, to the best of our knowledge, previously possible. COMET currently comprises 44 activating and 12 inhibitory zinc-finger TFs and 83 cognate promoters, combined in a framework that readily accommodates new parts. This system can tune gene expression over three orders of magnitude, provides chemically inducible control of TF activity, and enables single-layer Boolean logic. We also develop a mathematical model that provides mechanistic insights into COMET performance characteristics. Altogether, COMET enables the design and construction of customizable genetic programs in mammalian cells.

## Abstract (web summary)

Engineering mammalian cellular functions requires a toolkit of orthogonal and well-characterized genetic components. Here the authors develop COMET: an ensemble of transcription factors, promoters, and accompanying models for the design and construction of genetic programs.

## Introduction

The construction of synthetic genetic programs has emerged as a powerful approach for investigating signaling and regulatory networks^1^ and for engineering cell-based therapeutic and diagnostic devices^2,3^. Applications in mammalian cells often involve designing new ways for cells to sense and respond to internal states or environmental cues. Most programs utilize transcriptional regulation, and while large libraries of components such as transcription factors (TFs) and promoters have been developed for prokaryotes^4^, a dearth of analogous parts for mammalian systems currently limits both fundamental research and applications in medicine.

Early synthetic TFs used in eukaryotic cells employ the bacterial tetracycline-responsive repressor TetR^5,6^ or yeast Gal4^7^, and these proteins remain workhorses. New TFs have expanded the pool of orthogonal regulators through programmable DNA recognition, including zinc-finger (ZF)-TFs^8,9^, transcription activator-like effectors (TALEs)^10–12^, dCas9-TFs^13^, and TetR family regulators^14^. ZF-TFs are especially attractive for building a toolkit for transcriptional control, as they are the smallest of these new TFs, affording space for more complex genetic programs under constraints such as gene delivery vehicle cargo limits.

An ideal transcriptional toolkit would include well-characterized TFs and promoters; a physical understanding of how design choices impact performance characteristics; and a quantitative framework that describes how such biological parts may be combined to produce intended behaviors. Such a toolkit should include multiple orthogonal activating and inhibitory TFs; sets of TFs and promoters that enable one to experimentally scan through values of a given performance characteristic; and modularity in TF and promoter design to enable swapping and expansion of the toolkit and interfacing with other biological parts.

To address these needs, we report the COmposable Mammalian Elements of Transcription (COMET)—an ensemble of engineered promoters and modular ZF-TFs with tunable properties. We incorporate into COMET a panel of 19 TFs that were originally developed in yeast^15^ using designed ZF domains^16^. We characterize new promoters and then append new functional domains onto the ZFs. In doing so, we elucidate design rules for utilizing TFs and promoters to build gene expression programs exhibiting customizable activation, inhibition, small molecule-responsiveness, and Boolean logic in mammalian cells, and we develop a mathematical model to describe the properties of these genetic parts and programs.

## Results

### Identifying promoter design rules in mammalian cells

**Fig. 1:**
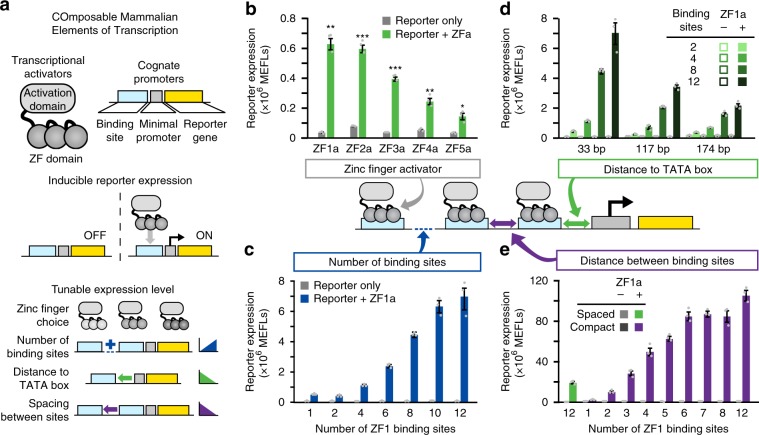
Investigation of COMET promoter design rules. **a** The schematic shows the modular, tunable features of COMET TFs and promoters. **b** Five ZFa with different ZF domains all induced reporter expression (one-tailed Welch’s *t*-test: *p *<* 0.05, **p *<* 0.01, ***p *<* 0.001). **c** Increasing the number of ZF binding sites increased the level of gene expression in the presence of ZFa (ANOVA *p* < 0.001) but not without ZFa (ANOVA *p* *=* 0.24). Reporter expression increased significantly from 6 to 8 and from 8 to 10 binding sites but not on either side of this range (Tukey’s HSD test with α = 0.05). **d** Moving the ZF binding site array farther upstream of the TATA box reduced reporter expression (two-factor ANOVA *p* < 0.001), and arrays with more binding sites showed more substantial decreases in reporter expression. **e** Compaction of ZFa binding sites enhanced ZFa-induced reporter expression, for an equivalent number of ZF binding sites (one-tailed Welch’s *t*-test, *p* = 0.002), and across compact promoters, ZFa-induced reporter expression increased with the number of binding sites (ANOVA *p* < 0.001). Reporter expression increased significantly from 2 to 3, 3 to 4, 5 to 6, and 8 to 12 binding sites (Tukey’s HSD test with α = 0.05). Experiments were conducted in biologic triplicate, and data were analyzed as described in Methods. Error bars represent the S.E.M. Source data are provided in the Source Data file.

In nature, TFs based on ZF domains coordinate diverse functions^17^. For example, the evolutionarily ancient and widely expressed SP1 contains three Cys2-His2-type ZF motifs (generally considered a minimal ZF domain), and SP1 binding sites appear as tandem arrays in genes regulating cell growth, apoptosis, and immune function, as well as in compact, dynamically regulated viral promoters such as the long terminal repeat of HIV^18^. To begin developing a toolkit for constructing transcriptional programs from basic parts, we first considered five synthetic ZF domains characterized in yeast by Khalil et al.^15^ and investigated whether these tools could be adapted to regulate transcription in mammalian cells. In this mammalian library, each TF comprises a ZF DNA-binding domain fused to the VP16 activation domain (AD), forming a ZF activator (ZFa) that recruits RNA polymerase II (RNAPII) and induces transcription^19^. A new cognate promoter was generated for each ZFa by placing one ZF binding site upstream of the YB_TATA minimal promoter (Fig. 1a), which confers low background and inducible expression in several cell types^20,21^. All five ZFa induced expression from their cognate reporters between 4 and 17-fold above background (ZFa-independent expression) (Fig. 1b, Supplementary Fig. 1a). Interestingly, the rank order of the magnitudes with which these ZFa induced their cognate reporters differed from that observed in a similar system in yeast^15^.

Initial reporter output was relatively dim—on the order of 10^5^ Molecules of Equivalent Fluorescein (MEFLs, an absolute unit of fluorescence^22^) per cell—so we explored strategies for building stronger inducible promoters. An established principle is that inducible gene expression increases with the number of TF binding sites, so we tested a panel of ZF1a-responsive promoters containing multiple ZF1 sites in an array upstream from the minimal promoter (Fig. 1c, Supplementary Fig. 1b). In general, ZF1a-inducible reporter expression increased with the number of sites while background was unaffected. The ZF1 promoter with 12 sites (ZF1x12) yielded 113-fold induction—approximately 12 times greater than the ZF1x1 promoter.

We hypothesized that expression might be influenced by the distance between the ZF binding site array and the TATA box, either favoring or blocking interactions with RNAPII, as was observed previously with synthetic promoters^23^. To investigate, we constructed promoters with the binding site array moved from 33 bp upstream of the TATA box (the original position) to 117 bp or 174 bp upstream. Overall, increasing spacing led to decreased expression, and this effect was especially pronounced for promoters with many sites (Fig. 1d). Thus, when ZFa bind farther from the promoter, the AD is seemingly too distant to contribute to transcriptional activation. This mechanism would also explain diminishing returns observed when adding sites to large arrays (Fig. 1c). In summary, increasing ZF-TF binding site count enhances gene expression, but only if the sites are near the TATA box.

Given these findings, we next investigated whether compacting binding sites near the minimal promoter could potentiate transcriptional output. Our initial constructs had 16–38 bp spacers between each 9 bp binding site. To generate a more compact structure, constructs were generated with 6 bp spacers, such that ZFa would bind 15 bp apart in a rotating configuration around the DNA, as one turn of the double helix is 10.5 bp. We hypothesized that this configuration could avoid steric occlusion while increasing the local concentration of ZFa. A panel of compact promoters was generated, each containing 1–12 binding sites in an array beginning 33 bp upstream of the TATA box. These new promoters yielded strong output and 360-fold induction over background (Fig. 1e, Supplementary Fig. 1c). The output of the strongest compact promoter (ZF1x12-C) was over five times greater than that of the comparable spaced promoter (ZF1x12-S). Background remained low across all constructs. Several of the strongest promoters exhibited mild squelching—a phenomenon in which inducing the expression of a TF (here, ZFa, which is expected to induce expression of the EYFP reporter) causes unexpected diminishment in the expression of a gene (here, the constitutively expressed EBFP2 transfection control) by competing for a limited pool of cellular resources that carry out transcription^19,24^. Here, squelching is apparent when cells with high EYFP expression have lower EBFP2 expression than do cells with lower EYFP expression (Supplementary Fig. 1c). Thus, COMET ZFa and promoters can be potentiated until they saturate the cellular capacity for transgene expression^25^, and one can use simple rules to titrate transcriptional output.

### Elucidating mechanisms of COMET gene expression

**Fig. 2:**
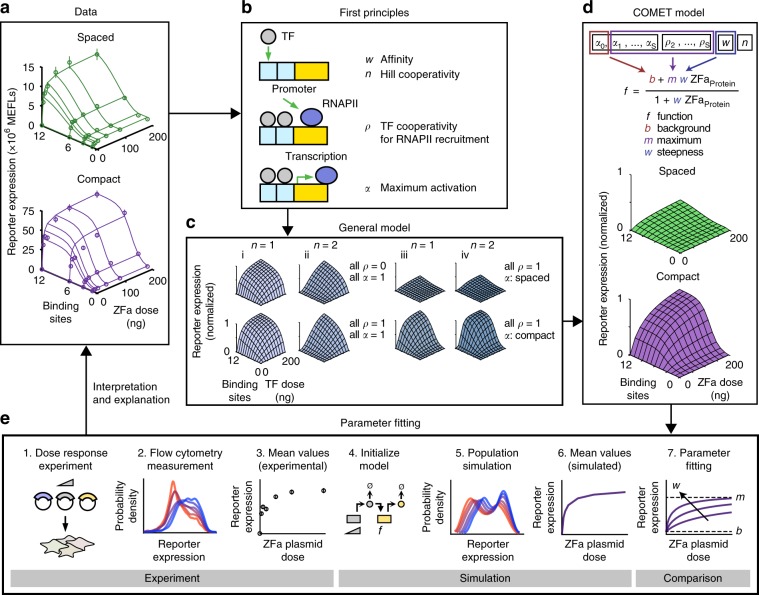
A model for COMET-mediated gene regulation. This figure summarizes the process of model development, refinement, and fitting. **a** The COMET model (model outputs are represented by the lines on each plot) explains experimentally observed trends (circles) for reporter expression as a function of ZFa dose and promoter features. This model uses a fitted response function for ZFa-induced gene expression (discussed in **b**–**e**) and simulates a cell population to account for variation in gene expression (Supplementary Fig. 3); lines depict the average outcome for the population. The experiment was conducted in biologic triplicate, and data were analyzed as described in Methods. Error bars represent the S.E.M. **b** We started with a detailed model of transcriptional activation in which reporter expression depends on TF concentration, a metric related to TF-DNA-binding affinity (*w*), TF-DNA-binding cooperativity (*n* = 1 for non-cooperative, *n* > 1 for cooperative), RNAPII recruitment cooperativity by each multiple-TF configuration at a promoter (*ρ* = 0 for non-cooperative, *ρ* > 0 for cooperative), and maximum promoter activation by each configuration (0 ≤ *α* ≤ 1). **c** This model yielded four types of landscapes (i–iv) under different assumptions, and two representative examples of each type are shown. COMET most closely resembles (iii). **d**, **e** A model that represents ZFa-induced reporter expression by a response function was used to fit the data in (**a**) (the workflow for parameter estimation is depicted in **e**). The terms in this concise model can be related to terms in the mechanistic model. Landscapes in (**c**,**d**) are simulations of a single cell (homogenous model), and those in (**a**) are simulated mean values for a heterogeneous population. The outputs of this final fitted model are represented alongside experimental data in (**a**). Source data are provided in the Source Data file.

Several observations prompted investigation into the COMET mechanism. At high doses of ZFa plasmid, reporter output plateaued at different levels depending on promoter architecture (Fig. 2a, Supplementary Fig. 2a–c). This plateau did not increase by switching minimal promoters, although some choices led to higher background (Supplementary Fig. 2d). Reporter expression increased with total plasmid dose (while holding the ratio of the ZFa plasmid and reporter plasmid constant), suggesting that transcription, and not translation, limits reporter output (Supplementary Fig. 2e).

To help elucidate the mechanisms by which COMET operates, we developed a mathematical model of this system. As summarized in Fig. 2 and detailed in Methods, we first considered mechanistic steps of gene expression, wrote equations capturing these steps (writing such equations is tantamount to formulating a hypothesis as to how gene expression operates), identified a formulation consistent with experimental observations, and simplified this representation by removing details not required to describe observed trends in order to generate a concise model. Finally, we fit parameters of the concise model to data in order to quantitatively describe experimental observations. We hypothesized that this process should generate a set of experimentally grounded parameters representing interpretable features of TF-promoter activity. Throughout, our goal was not to predict TF or promoter sequences de novo, but rather to describe and provide insight into observed trends. The explanatory value of such a model often exceeds insights that are accessible by intuition alone, and ultimately this framework could be used to design new genetic functions based upon COMET parts.

We initiated this process by using first principles to produce a detailed model with features of transcriptional control^26^ including physical and functional interactions between the promoter, TFs, and proteins like RNAPII (Fig. 2b, Methods). This detailed model relates transcriptional output to TF concentration, TF-DNA-binding affinity, TF-DNA-binding cooperativity, RNAPII recruitment cooperativity, and maximum promoter activation. We then generated a series of theoretical landscapes analogous to the experimental landscapes in Fig. 2a, varying parameters across a biologically reasonable range, and observed that the landscapes fell within one of four categories defined with respect to the concavity and sigmoidicity of cross-sections along each axis (Fig. 2c). The experimental data most closely resembled case (iii), indicating that TF-DNA-binding is non-cooperative, but RNAPII recruitment is cooperative, and the maximum transcription rate (at a high ZFa dose) increases with both the number and compactness of binding sites. Therefore, the enhanced potency of the compact promoters stems from the cooperative recruitment of transcriptional machinery.

Based upon the observed ZFa dose response profiles (Fig. 2a) and these insights, we proposed a concise response function to represent the rate of transcription (*f*) as a function of ZFa dose with three parameters: background transcription (*b*), a steepness metric (*w*) related to TF-DNA-binding affinity, and a metric for maximum transcription (*m*) (Fig. 2d, Methods). As indicated, the three parameters in this concise response function can be related to the additional parameters in the original detailed representation. For a given ZFa-promoter combination, *m* is experimentally determined and is based upon the number and spacing of binding sites in the promoter, and *b* is determined based on reporter expression without ZFa; *w* can be fit to ZFa dose response data by our previously developed method that improves parameter estimation by accounting for variation in gene expression^27^ (Fig. 2e, Supplementary Fig. 3a–c; fitted parameters are listed in Supplementary Tables 1 and 2). Simulated data from the calibrated model provided close agreement with the experimental data, demonstrating that a concise representation can be used to analyze and describe COMET-mediated gene expression.

Comparison of the calibrated model and experimental data confirmed two trends that hold across conditions (Supplementary Fig. 3d). First, the dependence of relative reporter output on binding site number is independent of the dose of ZFa plasmid when the output is scaled to its maximum value in each binding site series. Second, the dependence of relative reporter output on ZFa dose is independent of the number of binding sites when the output is scaled to its maximum value in each dose series. Thus, inducible gene expression follows patterns that hold across various promoter designs and that are captured by a concise model. The occurrence of these patterns, when paired with the properties elucidated by the model, makes ZFa-induced gene expression readily interpretable and ultimately usable—these are desirable features for a transcriptional toolkit.

### ZFa library characterization and orthogonality

**Fig. 3:**
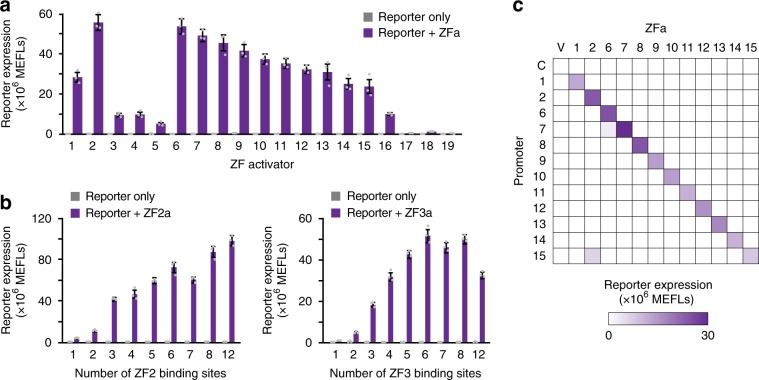
Characterizing an expanded panel of ZFa. **a** Nineteen ZFa were paired with cognate x6-C promoters, and all significantly induced gene expression (one-tailed Welch’s *t*-test all *p* < 0.02). **b** ZFa-induced gene expression increased with the number of binding sites, on compact promoters, for ZF2 (ANOVA *p* < 0.001) and ZF3 (ANOVA *p* < 0.001). **c** Investigating the orthogonality between the 12 strongest ZFa using x6-C promoters. Abbreviations: V (Vector control, no ZFa gene), C (Control reporter, no ZF binding sites). Experiments were conducted in biologic triplicate, and data were analyzed as described in Methods. Error bars represent the S.E.M. Source data are provided in the Source Data file.

Building upon our initial characterization of five ZFa (Fig. 1b), we evaluated whether 19 previously characterized ZFa^15^ could activate gene expression in mammalian cells. We observed that all ZFa drove transcription from their x6-C cognate promoters to varying extents (Fig. 3a, Supplementary Fig. 4a). Dose response profiles for the strongest 12 ZFa revealed a set of uncorrelated *m* and *w* values (Supplementary Table 1, Supplementary Fig. 5a–c). Additionally, the magnitude of induced reporter expression varied substantially between ZFa, which we hypothesized might be due to differential ZF affinity for binding cognate DNA sequences. Since the base pair upstream and base pair downstream (flanking nucleotides) of each 9 bp binding site affect ZF affinity^28^, we revisited promoters for two ZFa with contrasting outcomes in Fig. 3a (ZF2a for high expression and ZF3a for low expression) and observed that changing the flanking nucleotides significantly affected outcomes (Supplementary Fig. 5d). In both cases, changes guided by prior knowledge^15,28^ increased transcriptional activation, and thus choice of flanking nucleotides can be used to tune transcriptional activation. To test whether the magnitude of reporter induction mediated by ZF2a and ZF3a depends on the number of binding sites in a manner similar to that observed for ZF1a (Fig. 1e), we varied the number of sites using compact promoters, and observed a similar trend for up to eight sites (Fig. 3b). Interestingly, there was a small decrease in reporter expression as the number of binding sites increased from 6 to 7 for both ZF2 and ZF3. It is possible that some promoter architectures, such as ZFx7-C, involve mechanisms that result in slight deviations from overall trends.

To test whether ZFa-mediated activation of cognate promoters is orthogonal across ZFa-promoter combinations, we performed a series of pairwise evaluations using the twelve strongest ZFa and x6-C reporters. The highest expression from each promoter was observed with its cognate ZFa (Fig. 3c, Supplementary Fig. 5e). Of the 132 pairs of ZFa and non-cognate promoters, 80% showed less than 1% of the maximal expression from that promoter (i.e., off-target activation), and 97% showed less than 3% off-target activation. The highest off-target activation of a ZFa/non-cognate promoter pair (ZF2a/ZF15x6-C at 75%) may be explained by the similarities in the binding site sequences for ZF2 and ZF15 (7 of 9 bp in common). However, such sequence similarities were not noted for the next three highest off-target combinations (ZF6a/ZF7x6-C at 10%, ZF7a/ZF15x6-C at 6%, and ZF7a/ZF9x6-C at 4% off-target activation). Overall, COMET ZFa are generally orthogonal from one another and are thus well suited to composing genetic programs requiring multiple independent transcription units.

### Tuning transcription through protein engineering

**Fig. 4:**
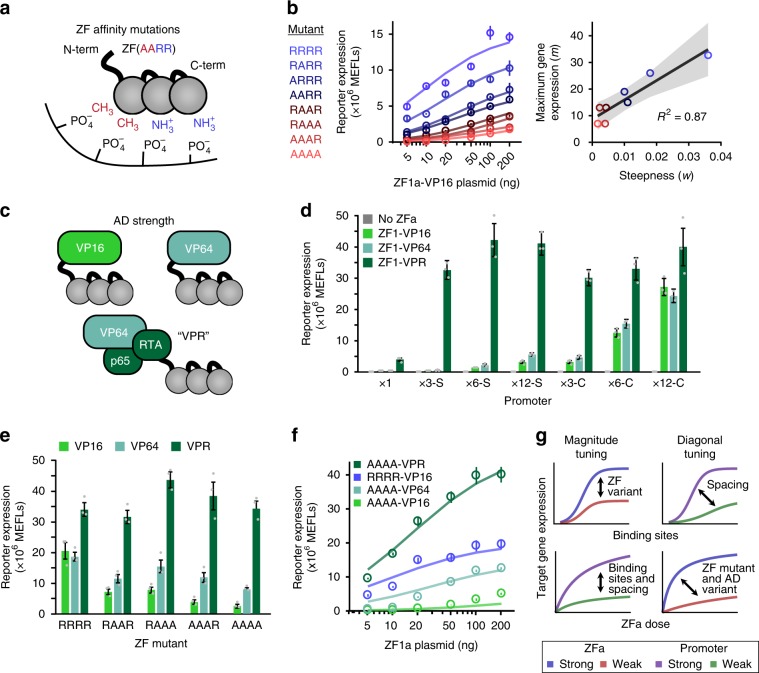
Tuning transcription through ZF mutants and AD variants. **a** The cartoon illustrates arginine-to-alanine (R-to-A) mutations in the ZF domain, which decrease the DNA-binding affinity. **b** Left: ZF mutations modulate the steepness and the maximum of the ZFa dose response profile. Circles represent experimental data and solid lines represent fitted response function models. Right: correlation between *m* and *w* parameters across mutants. The regression line is *m* = 7.3 × 10^2^*w* + 8.6, and the shaded region is the 95% confidence interval (one-tailed permutation test *p* < 0.001). **c** The cartoon depicts evaluated ADs. **d** Effects of AD on inducible reporter expression with different promoters. Gene expression varied with the choice of promoter (two-factor ANOVA *p* < 0.001) and choice of AD (*p* < 0.001), and an interaction was observed between these two variables (*p* < 0.001). **e** Combined effects of AD variants and ZF mutations were identified. Gene expression was affected by both the ZF mutations (two-factor ANOVA *p* *<* 0.001) and the AD (*p* < 0.001), with an interaction seen between these two variables (*p* < 0.001). Each mutant ZFa induced more reporter expression with VP64 than with VP16 (one-tailed Welch’s *t*-test, all *p* < 0.05) and with VPR than VP64 (one-tailed Welch’s *t*-test, all *p* < 0.01). All VPR-ZFa induced similar expression regardless of the use of a WT or mutant ZF (Tukey’s HSD test with α = 0.05). **f** The choice of AD affects the steepness and the maximum of the dose response. Circles represent experimental data and solid lines represent fitted response function models. **g** The cartoon summarizes expected trends in output gene expression that result from tuning each modular feature of the ZFa and promoters. These design choices can produce either a vertical shift or diagonal shift in response profiles with respect to the number of binding sites and the dose of ZFa. Experiments were conducted in biologic triplicate, and data were analyzed as described in Methods. Error bars depict S.E.M. Source data are provided in the Source Data file.

Having explored strategies for tuning gene expression by promoter engineering, we next investigated two strategies for tuning via protein engineering: altering the affinity of the ZF for the DNA and altering the strength of the AD. For the first strategy, we mutated four arginine residues in the ZF that interact with the DNA backbone (Fig. 4a). Arginine-to-alanine substitutions in these positions ablate favorable charge interactions between the ZF and negatively charged phosphates in the DNA backbone and decrease ZF1a-induced expression in yeast^15,29,30^. As expected, ZFa-mediated gene expression decreased with an increasing number of such substitutions (Fig. 4b, Supplementary Fig. 6a, b). Interestingly, while changing the promoter architecture affected only the maximum transcription (*m*) (Fig. 2), ZF mutations affected both the maximum transcription and the relative steepness of the ZFa dose response curve (*m* and *w*). Additionally, the changes in these values were correlated, revealing an axis along which ZFa R-to-A mutations tune TF strength. This result differs from our previous analysis of ZF domain choice, which affected *m* and *w* in an uncorrelated manner (Supplementary Fig. 5c). R-to-A mutations decreased ZFa-induced transcription in a manner that was similar across various numbers of binding sites in the promoter (Supplementary Fig. 6c), showing that this tuning can be applied across a variety of promoters. For the second tuning strategy, we tested two ADs in place of VP16: VP64^31^ and VPR^32^ (Fig. 4c). When fused in place of VP16, VPR produced the highest expression across several promoters, and VP64 was modestly stronger than VP16 in some cases (Fig. 4d). The relative effect of AD choice diminished as the number of ZF binding sites increased, suggesting that cooperative transcriptional activation by multiple weakly activating TFs (e.g., those based upon VP16), can approach the same magnitude of transcriptional activation mediated by fewer potently activating TFs (e.g., those based upon VPR). Replacing the AD on four other ZFa led to similar results (Supplementary Fig. 6d). Overall, these observations support the utility of multiple-TF engineering strategies for tuning gene expression.

To explore interactions between the two TF protein engineering strategies, we investigated whether stronger ADs could enhance gene expression conferred by TFs with low-affinity ZFs. As ZF binding affinity decreased, ZFa-mediated gene expression decreased substantially with VP16, yet only moderately with VP64 and not at all with VPR (Fig. 4e). We then compared the dose response for the weakest-binding ZFa mutant (AAAA) with each AD to the VP16 ZFa bearing a wild type (WT) ZF domain (Fig. 4f, Supplementary Fig. 6e). As AD strength increased, both *m* and *w* increased, as was observed when varying DNA-binding affinity. Although the two domains of a ZFa are physically modular, since they affect the same parameters in the response function, we find that the domains are functionally intertwined. Therefore, the two TF protein engineering strategies should be considered jointly when tuning a ZFa. In summary, our observations illustrate how gene expression can be tuned through selection of physical features—ZF domain choice, mutations that affect DNA-binding affinity, AD choice, and the number, spacing, and arrangement of binding sites in the promoter—and together this ensemble of designs provides a variety of realizable response profiles (Fig. 4g, Supplementary Fig. 6f).

### Design of inhibitory TFs

**Fig. 5:**
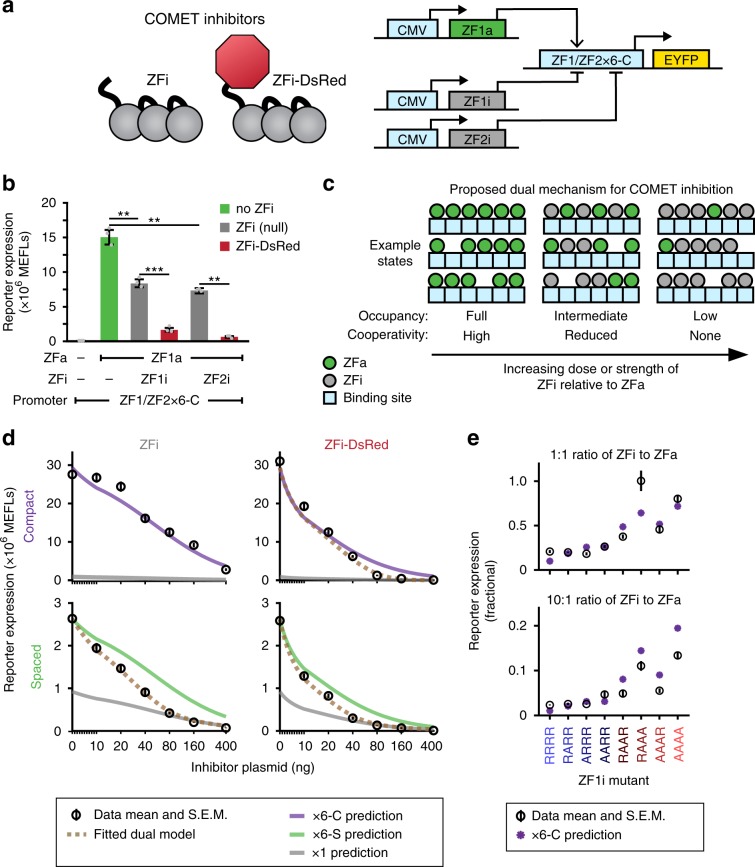
Transcriptional inhibition. **a** The schematic depicts two types of inhibitors that were evaluated. A ZF1/ZF2x6-C hybrid promoter is activated by ZF1a and inhibited by ZF1i or ZF2i. **b** ZFi and ZFi-DsRed differentially inhibit transcription (one-tailed Welch’s *t*-test: **p < 0.01, ***p < 0.001). **c** The cartoon summarizes the proposed conceptual model of ZFi-mediated inhibition. Within each cell, a promoter can occupy states with different configurations of ZFa and ZFi. Several example states are shown for three conditions of increasing dose or strength of inhibitor (i.e., DNA-binding affinity) relative to activator. **d** ZFi and ZFi-DsRed differ from standard competitive inhibitors. Predictions for competitive inhibition alone, for various promoter configurations, are depicted with solid lines (Methods). COMET inhibitors track the dotted lines, which represent fits to the dual mechanism model, except in the case of ZFi paired with x6-C, which tracks the competitive inhibition-only prediction. Each condition uses ZF1a at a dose of 40 ng. *X*-axes are scaled linearly from 0 to 10 ng and logarithmically above 10 ng. **e** Measured and predicted reporter expression were compared for a panel of ZFi mutants. Each condition uses ZF1a(RAAR) at a dose of 40 ng and the ZF1x6-C compact promoter. Experiments were conducted in biologic triplicate, and data were analyzed as described in Methods. Error bars represent the S.E.M. Source data are provided in the Source Data file.

Inhibitors comprise a key component of a versatile TF toolkit. We hypothesized that removing the AD from the ZFa would result in an inhibitor that binds DNA without inducing transcription (ZF inhibitor, ZFi) (Fig. 5a). We built a promoter with six compact binding sites for ZF1 and in which each ZF1 site overlapped with a ZF2 site to allow for pairwise testing of ZFi and ZFa with fully or partially overlapping sites (Supplementary Fig. 7a). Co-expressing ZF1a with ZF1i or ZF2i (equimolar plasmid doses) resulted in a ~50% decrease in inducible expression compared to ZF1a only, and inhibition mediated by partially overlapping ZF2i resembled that mediated by fully overlapping ZF1i (Fig. 5b, Supplementary Fig. 7b). This pattern held across various ZFi doses, and nearly complete inhibition was attained at high ZFi doses (Supplementary Fig. 8a). We hypothesized that transcriptional inhibition could be increased by incorporating a bulky domain to sterically block ZFa from binding adjacent sites in the promoter or to block the recruitment of RNAPII or associated factors. To test this hypothesis, we fused DsRed-Express2 (abbreviated throughout as DsRed) to the ZF domain. Co-expression of ZFi-DsRed and ZFa (equimolar plasmid doses) reduced reporter expression by 90–95%, and at higher ZFi-DsRed doses the inhibition was essentially complete, even when using stronger promoters based upon the CMV minimal promoter (Fig. 5b, Supplementary Fig. 8b, c). Therefore, the choice of a fusion partner affords an additional design handle for substantially tuning ZFi performance characteristics.

To help understand the mechanism of ZFi-mediated transcriptional inhibition, we considered that within each cell, promoters occupy an ensemble of states that depend on the promoter architecture and the ZFa and ZFi that are present (Fig. 5c). As the relative dose of ZFi to ZFa increases, the distribution of the ensemble should shift toward states that are more inhibited; a trend towards more inhibition should also occur by increasing the relative DNA-binding affinity of the ZFi versus that of the ZFa. Given our understanding of ZFa-mediated transcriptional activation, we speculated that the inhibitors should act via a dual mechanism with these properties: (i) competitive inhibition: since each site in the promoter can accommodate at most one TF, the binding of an inhibitor should prevent the binding of an activator; and (ii) decreased cooperativity: since inhibitors intersperse between activators, the spacing between activators should widen, and the effective *m* should resemble that of a promoter with lower cooperativity.

To explore this proposed mechanism of inhibition, we developed a model that describes the activity of ZFa and ZFi at a single-site promoter by representing physical interactions without a response function (Supplementary Fig. 8d, Methods). Simulated trends for ZFa dose responses with perturbations to DNA-binding affinity broadly agreed with experimental data (Supplementary Fig. 8e, Fig. 4). However, simulated trends for ZFa dose responses for varying AD strengths (at the simulated single-site promoter) differed qualitatively from the trends observed experimentally for a multi-site promoter (Fig. 4f). The difference in outcomes for the single-site and multi-site cases is consistent with our expectation that cooperative ZFa-mediated RNAPII recruitment would be observed only for the latter case (Fig. 2). Notably, the model also showed less responsiveness of reporter output to ZFi (at the simulated single-site promoter) than was experimentally observed for a multi-site promoter (Supplementary Fig. 8f), again suggesting that for multi-site promoters, ZFi can impair ZFa-mediated transcription by disrupting cooperative RNAPII recruitment.

To experimentally test the proposed dual mechanism of inhibition, we conducted dose responses for the ZFi and ZFi-DsRed inhibitors using the ZF1x6-S and ZF1x6-C promoters, with ZFa dose held constant (Fig. 5d). When ZFi was applied to the compact promoter, reporter expression matched the concise model for competitive inhibition alone. However, for the other three cases, observed reporter expression began to deviate with increasing doses of inhibitor, and by high doses it showed complete loss of cooperative RNAPII recruitment. The inhibitor dose at which the experiment began to deviate from the model was lower for ZFi-DsRed compared to ZFi and for spaced promoters compared to compact promoters. At intermediate doses of inhibitor, reporter expression ramped down toward single-site promoter behavior (Fig. 5c middle column, Fig. 5d dotted lines, Methods), and by high doses the ramp down was complete (Fig. 5c right column). The highest dose of ZFi-DsRed, used with the compact promoter, resulted in a profound 400-fold decrease in reporter expression. To further examine the case where the employed inhibitor did not disrupt cooperative RNAPII recruitment (i.e., ZFi used with the x6-C promoter), we paired a panel of ZFi varying in DNA-binding affinity with a reduced-affinity ZFa mutant (Fig. 5e). For all cases examined, ZFi-mediated inhibition was still predicted by competitive inhibition alone (Methods). We conclude that the compact promoter is more capable of cooperative RNAPII recruitment than is the spaced promoter, and that ZFi is a weaker inhibitor than is ZFi-DsRed, such that the dual inhibition mechanism applies to three of the four types of inhibitor-promoter pairings evaluated, and the pairing most responsive to inhibition is ZFi-DsRed with a compact promoter. Thus, the mechanism by which cooperative transcriptional machinery recruitment renders the compact promoter architecture highly activatable by a ZFa also causes such promoters to be substantially inhibited through disruption of this mechanism by a ZFi-DsRed.

### Genomic integration of COMET TFs

**Fig. 6:**
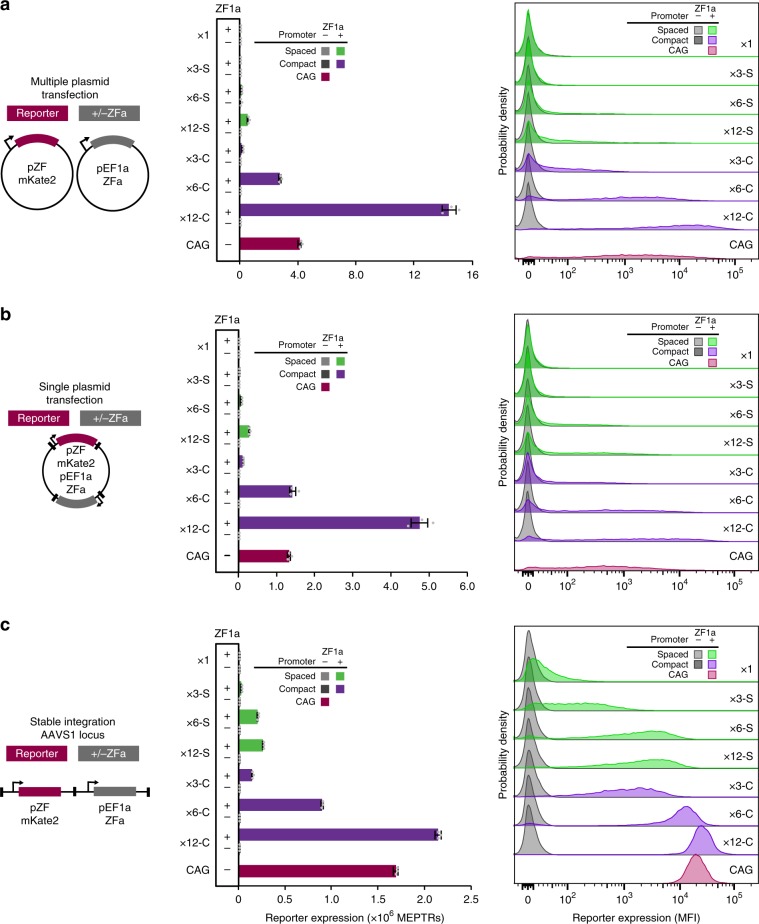
Characterization of promoter design rules in the genome. The cartoons summarize the systems used to evaluate promoter performance characteristics across three contexts: (**a**) multiple plasmid transient transfection, (**b**) single plasmid transient transfection, and **c** single-copy stable integration at a genomic safe harbor locus. The promoters included here comprise 1, 3, 6, or 12 ZF1 binding sites positioned using spaced or compact architectures upstream of the YB_TATA minimal promoter driving an mKate2 reporter gene. Constitutive EBFP2 was used as a transfection control in the transient transfection context and as a marker for genomic locus activity in the stable context. Bar graphs and histograms show reporter expression for EBFP2-expressing cells. In all contexts, ZFa-induced gene expression increased with the number of binding sites on spaced and compact promoters (ANOVA *p* < 0.00001). To profile the range of inducible expression conferred by each promoter, stable cell lines and transiently transfected cells were characterized using two distinct sets of flow cytometry settings (voltages), each of which was independently calibrated to yield comparable absolute fluorescence units (bar graphs). Experiments were conducted in biologic triplicate, and data were analyzed as described in Methods. Error bars represent the S.E.M. Source data are provided in the Source Data file.

Since some applications require stable integration of genetic programs, we investigated how COMET parts function upon stable integration into the genome, and in particular, whether COMET design rules gleaned from transient transfections might extend to performance in the genomic context. As a representative test set, we generated a panel of stable cell lines that each constitutively express a ZFa and contain one of several COMET promoters—varying the number of ZF binding sites and spacing between binding sites—that drive expression of a fluorescent reporter protein. To enable comparisons using a consistent site of genomic integration, we used site-specific Bxb1 recombinase-mediated integration into the AAVS1 safe harbor locus of HEK293FT landing pad cells^33^. In this process, COMET components were cloned into transcription unit positioning vectors (TUPVs) followed by one-step assembly into all-in-one integration vectors (IVs). The IVs used include a constitutive fluorescent protein marker and antibiotic resistance markers, a COMET promoter-driven mKate2 reporter, and either a constitutively expressed VP16-ZF1a or a blank control sequence (Supplementary Fig. 9a–c). Following gene delivery and selection (Supplementary Fig. 9d), we obtained cell lines that enable a comparison of COMET-driven gene expression in the stable genomic context (Fig. 6c, Supplementary Fig. 10c) to delivery by transfection of separate plasmids (Fig. 6a, Supplementary Fig. 10a) or transfection of all-in-one vectors (Fig. 6b, Supplementary Fig. 10b).

Overall, genomic COMET components drove gene expression following trends that are consistent with those observed in transient transfection: compact promoters drove more expression than did spaced promoters, and expression increased with the number of binding sites. Interestingly, for compact promoters, increasing the number of binding sites also led to more homogeneous reporter expression profiles spanning only a single order of magnitude—matching the tight distribution expected of a constitutively expressed gene in a landing pad^33^. For the strongest promoters (x6-C and x12-C), tight distributions of reporter expression contributed to high fold inductions (8000 and 14,000, respectively, compared to corresponding reporter-only cell lines). The promoter containing a single ZF1 binding site, placed in a favorable position with respect to the TATA box (Fig. 1d), did confer modest but significant gene expression compared to the control promoter lacking any ZF1 site (Supplementary Fig. 10d), although the expression induced by this ZFa from a x12-C promoter was 800-fold higher (Fig. 6c). Thus, COMET TFs can drive expression from either the genome or a plasmid, and the design rules used to tune expression in transient transfections may be transferrable, at least qualitatively, to the genomic context.

### Design and evaluation of small molecule-responsive TFs

**Fig. 7:**
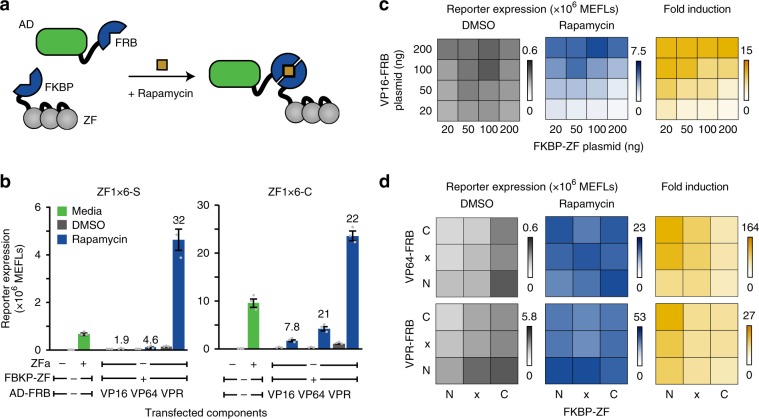
Engineering small molecule-responsive TFs. **a** The cartoon illustrates chemically responsive control of gene expression using rapamycin-inducible ZFa (RaZFa). **b** The effects of promoter architecture and AD on RaZFa performance were evaluated. For all RaZFa on both promoters, reporter expression was significantly higher with rapamycin than DMSO (one-tailed Welch’s *t-*test, all *p* < 0.05). Fold induction is shown above the rapamycin case for relevant conditions. **c** Gene expression in the absence of rapamycin was affected by VP16-FRB dose (two-factor ANOVA *p* *<* 0.001) and FKBP-ZF dose (*p* < 0.001), with no interaction between these variables (*p* = 0.14). Reporter expression after rapamycin addition was affected by VP16-FRB dose (two-factor ANOVA *p* < 0.001) and FKBP-ZF dose (*p* < 0.001) with a significant interaction between these variables (*p* < 0.001). **d** Effects of subcellular localization tags: N = nuclear, x = no localization, C = cytoplasmic. For VP64-based RaZFa, gene expression in the absence of rapamycin was affected by AD-FRB localization (two-factor ANOVA *p* = 0.01) and FKBP-ZF localization (*p* < 0.001), with no interaction between these variables (*p* = 0.39). For VP64-based RaZFa, gene expression after rapamycin addition was not affected by AD-FRB localization (two-factor ANOVA *p* = 0.26) but was affected by FKBP-ZF localization (*p* = 0.02), with an interaction (*p* = 0.001). For VPR-based RaZFa, gene expression in the absence of rapamycin was affected by AD-FRB localization (two-factor ANOVA *p* < 0.001) and FKBP-ZF localization (*p* < 0.001), with an interaction (*p* = 0.03). For VPR-based RaZFa, gene expression in the presence of rapamycin was affected by AD-FRB localization (two-factor ANOVA *p* < 0.001) but not by FKBP-ZF localization (*p* = 0.29), with no interaction (*p* > 0.05). Experiments in (**c**, **d**) use a ZF1x6-C promoter. Experiments were conducted in biologic triplicate, and data were analyzed as described in Methods. Error bars represent the S.E.M. Source data are provided in the Source Data file.

Chemical inducibility is useful for conferring external and temporal control over gene expression. We designed a small molecule-responsive ZFa by fusing FBKP and FRB domains, which heterodimerize upon exposure to rapamycin^34^, onto ZF and AD, respectively (Fig. 7a). We expected that without rapamycin, the ZF would bind DNA and not induce transcription, and that with rapamycin, FKBP and FRB would dimerize to reconstitute a functional ZFa. Indeed, rapamycin-activated ZFa (RaZFa) with each AD showed rapamycin-induced reporter expression (Fig. 7b, Supplementary Fig. 11a). Thus, COMET TFs can be adapted to achieve small molecule-induced gene expression.

We noted that fold increase in reporter output was lower for the RaZFa (± rapamycin) than for the ZFa (± ZFa). For the RaZFa using VP16, this effect was attributable to low induced reporter expression. We hypothesized that if FKBP-ZF were present in excess, it might competitively inhibit the reconstituted RaZFa from binding the promoter. To investigate, we varied the doses and ratios of RaZFa components (Fig. 7c, Supplementary Fig. 12a). High FKBP-ZF levels diminished expression as a ZFi would, and excess VP16-FRB increased inducible expression, resulting in high fold induction when paired with lower doses of FKBP-ZF. However, VP64-based RaZFa and VPR-based RaZFa were less affected by component ratios (Supplementary Fig. 12b, c). Thus, it appears that the relative weakness of VP16-mediated transcriptional activation makes VP16-based RaZFa more sensitive to excess FKBP-ZF.

Since high background in the absence of rapamycin limited the fold induction for VP64-based and VPR-based RaZFa, we investigated strategies to decrease background. VPR-FRB alone promoted a very low amount of reporter expression, and this background was greater in the presence of ZF-fusion proteins, even in the absence of rapamycin (Supplementary Fig. 12d), suggesting that the ZF can bind the promoter in such a way that transient promoter-AD interactions induce some transcription. To circumvent this putative undesired mechanism, we removed the nuclear localization signal (NLS) from each RaZFa component or replaced the NLS with a nuclear export signal (NES) (Fig. 7d, Supplementary Fig. 12e). For both VP64-based and VPR-based RaZFa, NES tagging of AD-FRB and NLS tagging of FKBP-ZF decreased background while conferring little effect on rapamycin-induced reporter expression, such that fold induction improved. To explain why the addition of NES to FKBP-ZF increased background, we hypothesize that while low levels of nuclear FKBP-ZF are sufficient to allow AD-FRB to drive transcription from the promoter, at higher nuclear levels the FKBP-ZF can act as an inhibitor. The decrease in background associated with the NES tag on AD-FRB was not due to decreased component expression (Supplementary Fig. 12f). Expression of VP64-FRB was low relative to other components, but increasing the dose of VP64-FRB plasmid—above levels used in Supplementary Fig. 12b—increased background and diminished inducible reporter expression (Supplementary Fig. 12g). Altogether, tuning these design variables led to improved rapamycin-inducible gene expression (greater fold induction) for each AD choice (Supplementary Fig. 13a), with responsiveness across several orders of magnitude of rapamycin concentration (Supplementary Fig. 13b) and yielding a useful system for chemically induced expression.

### Implementing Boolean logic with COMET

**Fig. 8:**
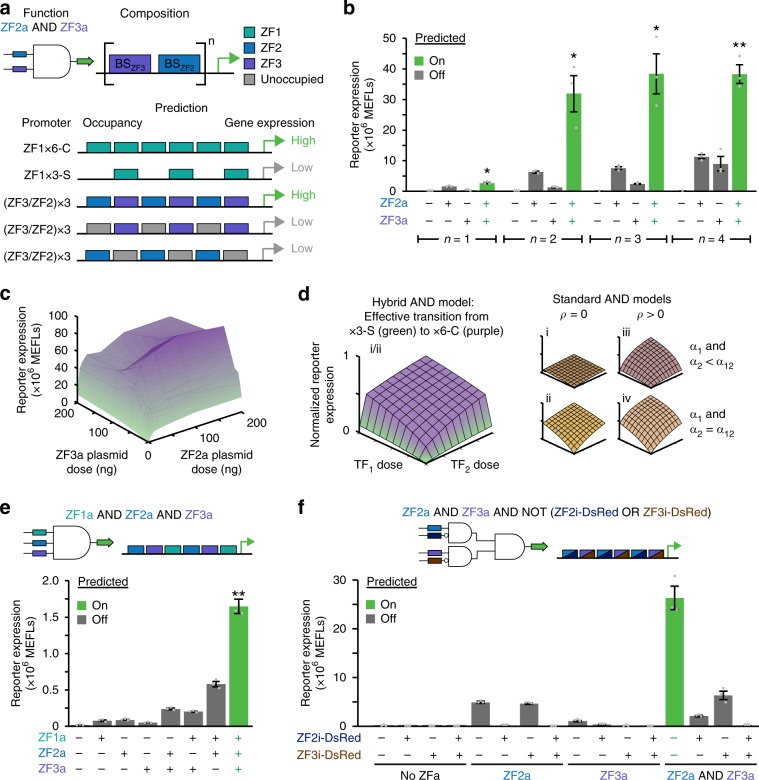
Composing Boolean logic. **a** The cartoon summarizes a strategy for single-layer, promoter-based logic gates with ZF-TFs. We hypothesized that AND gate promoters could be designed by using multiple repeats of a paired ZF3/ZF2 motif. Full occupancy of this promoter by both ZF2a and ZF3a mimics a fully occupied x6-C promoter, and partial occupancy (with either ZFa alone) mimics an x3-S promoter. Thus, there is a large increase in gene expression when the promoter is occupied by two types of ZFa compared to one type. **b** Candidate two-input AND gates were constructed using one to four repeats of paired binding sites in the promoter. AND gate behavior is considered significant if reporter expression with both ZFa is greater than the sum of reporter expression with each ZFa individually (one-tailed Welch’s *t*-test: *p < 0.05, **p < 0.01). **c** Two-input dose response for the AND gate with three repeats of paired binding sites. The landscape is shaded from green to purple to facilitate visualization in the z-axis direction. **d** A theoretical model of COMET AND behavior is compared with other models of transcriptional AND gates; the latter vary in whether activators have multiplicative cooperativity (*ρ*) and whether maximum activation (*α*) is equivalent for TFs individually and together (Methods). **e** A three-input AND gate was constructed using two repeats of a triplet binding site motif. AND gate behavior is considered significant if reporter expression with all three ZFa is greater than the sum of reporter expression with each ZFa individually, and also greater than the sum from each of the three combinations with two co-expressed ZFa and the other ZFa individually (one-tailed Welch’s *t-*test, **p < 0.01 for all four of these tests). **f** A four-input gate was constructed using the binding site arrangement shown. Experiments were conducted in biologic triplicate, and data were analyzed as described in Methods. Error bars represent the S.E.M. Source data are provided in the Source Data file.

Finally, we explored whether COMET could be used to encode Boolean logic functions within individual promoters. Our exploration of promoter architecture (Fig. 1c, e) suggested a strategy for designing hybrid promoters with alternating sites for combinations of ZFa to implement AND logic (Fig. 8a). We hypothesized that cooperative activation on compact promoters would occur only when both species of ZFa were present, conferring AND gate behavior. Synergistic activation arising from closely arranged TF binding sites has been used to build AND gates in mammalian genetic programs^23^, but arranging sites in alternating patterns does not necessarily guarantee the synergy required for an AND gate^27^. We tested promoters containing various pairs of ZF2 and ZF3 sites (Fig. 8b, Supplementary Fig. 14a). In each case, maximal reporter expression occurred when both ZFa were present, and this expression was greater than the sum of those induced by each ZFa individually—this defines AND gate behavior. For the three-pair hybrid promoter, AND gate behavior was observed even at low ZFa levels; 5 ng of each plasmid encoding ZF2a and ZF3a together produced more reporter expression than did 200 ng of plasmid encoding either ZFa alone (Fig. 8c, Supplementary Fig. 14b). The steep OFF-ON transition along the perimeter of the dose response landscape is due to the effective transition between x3-S and x6-C architectures—an advantageous behavior of COMET that differs from previously reported transcriptional AND gates utilizing tTA and Gal4 (Fig. 8d, Supplementary Fig. 14c, Methods)^27^.

We extended this hybrid promoter strategy to generate candidate three-input AND gates for ZF1a, ZF2a, and ZF3a. A promoter with one site for each ZFa did not produce AND gate behavior (Supplementary Fig. 14d), which is consistent with the expected similarity in reporter expression for promoters recruiting two versus three ZFa (Fig. 1c, e). However, a promoter with two sites per ZFa did produce AND gate behavior; reporter expression when all three ZFa were present was higher than the sum of the levels when any two ZFa were present plus the level conferred by the third (Fig. 8e). Thus, COMET’s modular features enable the composition of single-promoter AND gates.

Finally, we investigated whether inhibitors could be combined with activators to build complex logic functions using design rules elucidated in this study. As a test case, we designed a four-input logic function that takes both activators and inhibitors as inputs (Fig. 8f, Methods). We first characterized individual interactions between activators and inhibitors and found that ZF2i-DsRed and ZF3i-DsRed were the most effective at inhibiting expression (Supplementary Fig. 14e, f). In the full genetic program, all cases produced the expected outcomes (Fig. 8f, Methods). Thus, COMET components and design principles can be employed to compose complex functions with single-layer logic.

## Discussion

We anticipate that COMET will be a useful resource for building genetic programs. Currently, the engineering of mammalian cellular functions is slow and involves multiple iterations of the design-build-test-learn cycle. In prokaryotes, the design and construction of genetic programs have been streamlined by the development of large libraries of well characterized and orthogonal components in concert with computational tools such as Cello^35^. COMET similarly provides a large library of TFs and promoters with tunable features, and the characterization of these components provided a foundation for a mathematical model. We used the model to elucidate mechanisms by which the activators and inhibitors operate at promoters and fitted parameters to describe how these activities vary across the design choices examined. This integrated approach transcends the identification of general qualitative trends (e.g., that increasing the number of binding sites in a promoter generally increases inducible gene expression) to yield quantitative and often mechanistic understanding as to how design choices affect TF-promoter activity. This insight could not have been deduced from prior knowledge, including biophysical intuition or even characterization of similar ZFa and promoters in yeast^15^. Whether the design rules elucidated here ultimately enable large-scale, model-driven design is an important question worthy of subsequent investigation.

COMET comprises an extensible toolkit that readily accommodates new parts. The current library includes 44 activating and 12 inhibitory TFs and 83 cognate promoters. Of the 44 ZFa, 19 were ported from a toolkit originally characterized in yeast^15^ with only minor changes in the linker between protein domains. Generating the remaining activators and inhibitors involved combining ZF domains with functional domains. This highlights COMET’s modularity, in that new elements can be characterized, modeled (Fig. 2, Methods), and then utilized for customized gene regulatory functions.

Our combined experimental and computational investigation revealed properties and design rules that guide the use of COMET parts. By selecting TF-promoter combinations, one can select a magnitude of output gene expression from a range spanning three orders of magnitude. The design rules explain, at a high level, the functional consequences of choices such as ZF domain, mutations in the ZF domain that impact binding affinity, the AD, competition between activating and inhibitory TFs, and the number, spacing, and arrangement of binding sites in the promoter. COMET-mediated gene expression confers dose response landscapes that differ from those of tTA and Gal4^27^ and thus could be better suited for applications such as building hybrid promoters. COMET is also amenable to the incorporation of other functional modalities such as chemically inducible gene expression.

A key insight is that COMET promoter strength arises from cooperative recruitment of transcriptional machinery, which is an effect that varies with the number of and spacing between binding sites. This mechanism differs from that of previously characterized ZF-TF systems in which cooperativity is directly engineered into TFs through protein–protein interaction domains such as PDZ or leucine zippers^9,15,36^. While these previous strategies usefully enable the tuning of performance characteristics such as dose response curves, they are potentially limited by the availability, orthogonality (with respect to both synthetic and endogenous components), and geometric requirements of the protein–protein interaction domains employed. In contrast, the scalability of COMET thus far appears limited only by the availability of orthogonal ZFs; these domains can be constructed using technologies such as OPEN^16^ as well as other methods, and this remains an active area of research.

COMET’s promoter-based cooperativity is useful. First, it confers both low background expression and high fold induction, even though these two objectives typically present a trade-off^21^. Second, it enables the implementation of logic gates that have attractive features. Unlike other previously described logic gates that require different architectures for activation and inhibition^9^, a single COMET promoter can be used in either activating or inhibitory logic gates. Many gates function as predicted without extensive tuning (Fig. 8). These properties simplify the design process and enable integrating multiple inputs at a single promoter, ultimately decreasing the number of components required to construct genetic programs. Inhibitory COMET TFs modulate effective cooperativity to completely inhibit transcription (Fig. 5, Supplementary Fig. 8). This mechanism is fast and likely reversible, which could be advantageous over mechanisms that employ slower KRAB-mediated chromatin repression and subsequent reactivation^37^.

Another advantage of promoter-based cooperativity is that it should enhance the specificity with which ZFa activate target promoters. A limitation to the minimal three-finger ZF-TF strategy investigated here is that any single 9 bp ZF binding sequence might occur many times in a genome. However, the probability that two binding sites would occur at the same locus is unlikely, and the chance that three or more sites would co-occur is vanishingly small. Moreover, the potent activation reported in Fig. 1 also required the ZF binding array to be proximal to a transcriptional start site, which should further boost the distinction between on-target and off-target transcription. Indeed, in a genomic context (Fig. 6), although ZF1a drove modest expression from a x1 promoter (in which the ZF binding site was placed favorably close to the TATA box), the expression from a x12-C promoter was 800-fold greater. The protein engineering design rules elucidated here also suggest that specificity could be further increased, if desired, by the choice of AD and ZF domain. For example, selection of a weaker AD could necessitate that multiple ZFs bind in a compact configuration at a promoter in order to drive transcription (Fig. 4d). Reducing the affinity with which a ZF binds DNA could also be combined with selection of a weaker AD to shift the dose response curve, such that a promoter is activated only at high concentrations of ZFa (Fig. 4f). Thus, a potential advantage of pairing weaker ZFa with multi-site promoters is the possibility of dramatically boosting the effective specificity of the ZFa for driving transcription from a target promoter. Chromatin state, and thus cell type, likely impacts the trade-off between on-target and off-target gene regulation, and this question is worthy of exploration in future applications.

Several properties are not easily explained by simple design rules. It is not yet clear why some ZFa combinations exhibit limited crosstalk when no sequence similarity in ZF binding sites is apparent (Fig. 3c), though our empirical evaluation does identify how such crosstalk can be avoided. Also, some non-specific transcriptional activation was conferred by the most potent ADs (e.g., VPR) when ZF domains were separately expressed but not driven to physically associate (i.e., by addition of rapamycin), suggesting a noncanonical mechanism. Operationally, these phenomena present minor complications that can be circumvented by system selection and attentiveness to potential artifacts during development and design of new functions.

It will be interesting to evaluate how the trends observed here are conserved or diverge as the COMET toolkit grows and is applied to new applications. For example, we cannot predict a priori the magnitude of gene expression that a new ZFa will confer on its cognate promoter, nor can we predict orthogonality, but our analysis suggests that new parts may be screened, tuned, and combined following the same principles used in this study. We expect that the specific quantitative parameters determined in this study could be limited to the implementations used here, including the methods for DNA delivery and the cell type in which the characterizations were performed. However, since the fundamental mechanisms of transcription are maintained across contexts, we expect that the observed trends will extend across cell types and delivery methods. For instance, the rank order of promoter strength across the number of binding sites was conserved between transient transfection and genomic integration (Fig. 6). In practice, it is straightforward to test a focused library of parts to empirically identify which combinations provide the function needed for an application, and if needed, tune system performance using strategies described in this study.

A particularly exciting prospect is using COMET with other synthetic biology technologies. For example, COMET could be integrated into synthetic receptors that utilize orthogonal TFs as outputs, such as MESA or synNotch, to generate cellular programs for sensing, processing, and responding to environmental cues^27,38–40^. Alternatively, COMET could be used to regulate the expression of synthetic components, such as GEMS receptors which interface with endogenous regulation^41^. We expect that COMET will be useful for prototyping and implementing sophisticated cellular functions for both fundamental research and cellular engineering applications.

## Methods

### General DNA assembly

Plasmid cloning was performed primarily using standard PCR and restriction enzyme cloning with Vent DNA Polymerase (New England Biolabs (NEB)), *Taq* DNA Polymerase (NEB), Phusion DNA Polymerase (NEB), restriction enzymes (NEB; Thermo Fisher), T4 DNA Ligase (NEB), Antarctic Phosphatase (NEB), and T4 PNK (NEB). Golden Gate assembly and Gibson assembly were also utilized. Most plasmids were transformed into chemically competent TOP10 *E. coli* (Thermo Fisher) and grown at 37 ˚C, except for integration vectors, which were transformed into chemically competent Stable *E. coli* (NEB) and grown at 30 ˚C.

### Cloning strategy for COMET vectors

The COMET plasmids are in pcDNA-based backbones for high expression in HEK293FT cells. Restriction sites were chosen to allow for modular swapping of parts with restriction enzyme cloning. Furthermore, reporter constructs can be assembled by one-step Golden Gate reactions employing synthesized oligonucleotides. A complete list of all plasmids constructed for and utilized in this manuscript is available in Supplementary Data 1, and plasmid maps are available per Data Availability.

### Source vectors for DNA assembly

ZF-containing and VP16-containing vectors were a generous gift from Ahmad Khalil^15^. VP64 and VPR were sourced from SP-dCas9-VPR, which was a gift from George Church (Addgene plasmid #63798)^32^. DsRed-Express2 was obtained by site directed mutagenesis of pDsRed2-N1, which was a gift from David Schaffer (University of California, Berkeley). EBFP2 was sourced from pEBFP2-Nuc, which was a gift from Robert Campbell (Addgene plasmid #14893)^42^. EYFP, FKBP, and FRB were sourced from plasmids we previously described (Addgene plasmids #58855, #58877 and #58876, respectively)^38^. NanoLuciferase was synthesized as a GeneArt DNA String (Life Technologies/Thermo Fisher). The mMoClo (pLInk2, pLink4, and pLink8, Destination Vector, BxB1 Recombinase Expression Vector) plasmids were a gift from Ron Weiss^33^. The CHS4 insulator was sourced from PhiC31-Neo-ins-5xTetO-pEF-H2B-Citrin-ins, which was a gift from Michael Elowitz (Addgene plasmid #78099)^37^. The CAG promoter was sourced from pR26R CAG/GFP Asc, which was a gift from Ralf Kuehn (Addgene plasmid #74285)^43^. The SV40 minimal promoter was sourced from pYC0866 (4xHRE_minSV40-sfGFP-CMV_dsRed Exp), which was a gift from Yvonne Chen^21^. EF1α and TetON3G were sourced from pLVX-Tet3G (Clontech), and TRE3GV was sourced from pLVX-TRE3G (Clontech). Barcodes used for the TUPVs were designed by the Elledge lab^44^. BlastR was sourced from lenti dCAS-VP64_Blast, which was a gift from Feng Zhang (Addgene plasmid #61425)^45^.

### Plasmid backbones

All plasmid backbones are modified versions of the pcDNA3.1/Hygro(+) Mammalian Expression Vector (Thermo Fisher V87020).

To make pPD003, the SV40 promoter and Hygromycin resistance gene that it drove were removed, while leaving the SV40 origin of replication and SV40 poly(A) signal intact. Additionally, a sense mutation in the *AmpR* gene was introduced to remove a BsaI restriction site.

To make pPD005 (referred to as “pcDNA”), the BpiI site in the BGH poly(A) signal was mutated to enable Golden Gate reactions with BpiI, and the BsaI site in the 5’-UTR was mutated to enable Golden Gate reactions with BsaI. The BpiI site was in a region of the BGH poly(A) tail that when deleted does not alter the efficiency of the polyadenylation^46^.

### Template plasmids for ZF reporter plasmids

pPD027 (the first-generation ZF reporter template) was constructed by inserting a synthesized region (containing two BsaI sites for Golden Gate-mediated ZF binding site array insertion and a YB_TATA minimal promoter^21^) between the BglII and NheI sites and inserting EYFP between the NheI and NotI sites of pPD003.

pPD032 and pPD033, which are the templates for ZF reporters with the binding site array moved further upstream of the minimal promoter, were constructed by inserting spacer regions into the BamHI site between the ZF binding array insertion template and the YB_TATA minimal promoter. The spacer inserts were amplified by PCR from the region of pPD003 upstream of the CMV promoter prior to insertion. These three templates (pPD027, pPD032, and pPD033) were used to construct all spaced reporters shown in Fig. 1b–e and Fig. 2a.

pPD152 (the second-generation ZF reporter template) was constructed to enable multi-round insertion of larger ZF binding arrays using alternating rounds of Golden Gate with BsaI and BpiI. To do so, the region of pDPD027 between the AatII and NotI sites (the ZF binding array insertion site through the end of the EYFP coding sequence) was inserted between the corresponding sites of pPD005. pPD152 was used to make all of the ZF1 compact binding site reporters shown in Fig. 1 and Fig. 2a (including pPD290 (ZF1x6-C YB_TATA EYFP), which was used as the reporter plasmid in the majority of the experiments), and the logic promoters in Fig. 8.

pPD540 (the third-generation ZF reporter template) was constructed to swap the palindromic “sticky ends” (5′ or 3′ overhangs) of the ZF binding array insertion site to non-palindromic sticky ends. The use of palindromic sticky ends, which were originally designed to allow construction of ZF binding arrays with either Golden Gate or EcoRI and BamHI, risks the insertion of multiple copies of the same insert in Golden Gate reactions. This redesign enabled us to inset promoters of sizes that could not be cheaply synthesized as a single insert as multiple inserts in a single round of Golden Gate. This was accomplished by synthesizing a new upstream region (containing two BsaI sites for Golden Gate-mediated ZF binding site array insertion with non-palindromic sticky ends and a YB_TATA minimal promoter) and inserting this upstream region between the BglII and NheI sites of pPD152.

### Golden Gate assembly of ZF reporter plasmids

Golden Gate assembly^47^ was used to construct most of the reporter plasmids from a reporter template. Promoter insets were synthesized as 15–100 bp oligonucleotides (some promoters were synthesized as multiple inserts) by Integrated DNA Technologies or Life Technologies (Thermo Fisher). The coding and reverse strands were synthesized separately and designed to anneal, resulting in dsDNA with a 4 nt sticky end overhang on each side. The coding and reverse oligonucleotides were mixed (6.5 µL H_2_O, 1 µL T4 Ligase Buffer, 0.5 µL T4 PNK (10 U/µL; NEB), 1 µL of each 100 µM oligonucleotide) and phosphorylated at 37 °C for 1 h. They were then denatured at 95 °C for 5 min and cooled slowly to room temperature (here, approximately 22 °C) to allow for annealing. The mix was then diluted 50-fold to make a 200 nM stock or 500-fold to make a 20 nM stock. While we made most of the constructs with the 200 nM stock, we later discovered that the 20 nM stock resulted in higher-efficiency reactions.

BsaI Golden Gate reaction mixtures comprise 1 µL T4 ligase buffer, 1 µL 10× BSA (1 mg/mL), 0.5 µL BsaI-HF (20 U/µL; NEB), 0.5 µL T4 Ligase (400 U/µL; NEB), 10 fmol of vector, 1 µL of each insert (diluted to 200 nM or 20 nM), and water to 10 µL total volume. The reaction was incubated at 37 °C for 1 h, 55 °C for 15 min, and 80 °C for 20 min, and then cooled to room temperature. Up to 10 µL of reaction was immediately transformed into up to 50 µL of chemically competent Top10 *E. coli*. For reactions that did not yield many colonies on the first cloning attempt or did not produce colonies with the correct plasmids, the reaction conditions were changed to: 30 cycles of 37 °C for 1 min then 16 °C for 1 min, 55 °C for 15 min, 80 °C for 20 min, and cool to room temperature.

Some of the larger ZF binding site arrays were assembled through sequential rounds of alternating BsaI and BpiI Golden Gate reactions. BpiI Golden Gate reaction mixtures comprise 1 µL T4 ligase buffer, 1 µL 10× BSA (1 mg/mL), 0.4 µL BpiI-FD (Thermo Fisher), 0.4 µL µL T4 Ligase (400 U/µL; NEB), 10 fmol of vector, 1 µL of each insert (diluted to 200 nM or 20 nM), and water to 10 µL. The reaction was incubated at 37 °C for 30 min, 50 °C for 5 min, and 80 °C for 10 min, and then cooled to room temperature prior to transformation.

### Non-Golden Gate assembly of some ZF reporters

Although Golden Gate assembly was the primary strategy for cloning the promoters, the first-generation templates were not readily amenable to synthesis and insertion of ZF binding site arrays. Therefore, some spaced promoters with large numbers of binding sites used in Fig. 1c were constructed by PCR amplification of 1–8 binding sites from other reporter plasmids and insertion of these binding sites between the EcoRI and BamHI sites upstream of reporter constructs with 1–8 binding sites in the promoter. Likewise, the ZF reporters with ZF binding site arrays moved further upstream of the minimal promoter shown in Fig. 1d were constructed by PCR-amplifying the ZF binding site arrays from other constructs and inserting between the EcoRI and BamHI sites of pPD032 and pPD033.

Additionally, COMET reporter constructs were designed to include a limited set of minimal promoters; restriction enzyme cloning was employed to accomplish this as well. pPD1028 (ZF1x6-C SV40_Min EYFP) was cloned from pPD270, cut with XbaI and ApaI. Into this construct we inserted two fragments of DNA: SV40_min^21^ was PCR-amplified and cut with BsaI and ApaI, and EYFP was PCR-amplified from pPD270 and cut with BsaI and ApaI. pPD1029 (ZF1x6-C CMV_min EYF) was cloned from pPD270, cut with XbaI and ApaI. CMV_min was synthesized by IDT and placed upstream of an EYFP gene in a pcDNA-based vector. A fragment comprising CMV_min and EYFP was then PCR-amplified, digested with BsaI and ApaI, and inserted into the digested pPD270.

### Assembly of ZFa and ZFi

The first five ZFa tested in Fig. 1b were constructed by PCR-amplifying the ZFa sequence from^15^ (including the N-terminal 3x-FLAG tag, SV40 NLS, VP16 AD, and ZF) and inserting between the NheI site and NotI site of pPD005. Cognate ZFi were constructed by whole-plasmid PCR-mediated deletion of the VP16 AD. During the AD deletion process, BamHI and KpnI sites were added between the SV40 NLS and the ZF, which were later used to insert a PCR-amplified DsRed-Express2, thereby creating cognate ZFi-DsRed. Subsequent ZFa (i.e., any new ZFa tested in Fig. 3a) were constructed by replacing DsRed-Express2 with a PCR-amplified VP16 (BamHI/KpnI) and replacing the ZF domain with a PCR-amplified ZF domain (KpnI/NotI) from^15^.

### Assembly of ZF mutants

ZFa mutants were synthesized as multiple sets of complementary oligonucleotides, which were annealed and then inserted via Golden Gate assembly into a vector designed to encode ZFa upon insertion of all inserts. Reactions were performed with BpiI as described in *Golden Gate assembly of ZF reporter plasmids*. ZFi mutants were generated by whole-plasmid PCR-mediated deletion of the VP16 AD.

### Assembly of RaZFa

RaZFa components were constructed by multi-step restriction enzyme-based cloning. The SV40 NLS was part of the original ZFa constructs^15^, and the NES sequence was obtained from^48^.

### Gibson assembly

Gibson assembly^49,50^ was used to specify ADs on ZF1a. Gibson reactions were performed by PCR addition of homology arms onto the target DNA. Components were mixed together: 17 fmol of backbone, 51 fmol of each insert, 7.5 μL of Gibson Master Mix, and water to 10 µL. 7.5 µL of Gibson Master mix contains 2 μL 5× isothermal reaction buffer (0.5 M Tris-HCl pH 7.5, 0.05 M MgCl_2_, 1 mM dNTP, 5 mM NAD, 0.05 M DTT), 0.04 U T5 exonuclease, 0.25 U Phusion DNA Polymerase, and 40 U *Taq* DNA Ligase (NEB) in water. The reaction was incubated at 50 °C for 1 h, and 5 µL was transformed into chemically competent Top10 *E. coli* (Thermo Fisher). In subsequent cases, ADs were moved onto other ZF by restriction digest.

### Construction of plasmids for mMoClo

We made several changes to the mMoClo plasmids originally described^33^ in order to incorporate them into the workflow for our laboratory, in which many constructs are prototyped using pcDNA-based expression vectors. Details can be found in Supplementary Fig. 9, Supplementary Data 1, 2. We modified the Destination Vector provided by the Weiss lab by adding two repeats of the CHS4 insulator into two places in the vector. The insulators upstream of the attB site are, upon genomic integration, inserted downstream of the LP, insulating the LP from the genome (and vice versa). The insulators downstream of the RB Globin polyA terminator of the puromycin resistance gene insulate this transcription unit from TU1. This new vector is termed pPD630 (Integration Vector). We cloned pLink1, pLink3, pLink5, pLink6, pLink7, and pLink9 site directed mutagenesis via whole-plasmid PCR of pLink2.

The TUPVs were cloned by making several alterations to pcDNA (pPD005), in three steps. In the first step, two repeats of the CHS4 insulator were placed downstream of the BGH polyA tail. Second, to enable Golden Gate cloning of the TUPV library, three pairs of BsaI sites were inserted into the vector with PCR. The first pair was upstream of the promoter, the second pair was inserted between the BGH polyA tail and the insulator, and the third pair was inserted downstream of the insulator. In the third reaction, three pairs of annealed oligonucleotides were inserted into these BsaI sites via a Golden Gate reaction. The first insert, to be placed upstream of the promoter, comprised a BpiI site, TUPV-specific sticky end, and TUPV-specific 5′ barcode (barcodes unique to each TUPV enable sequencing of the TUPV contents after TUPVs are combined into an integration vector). The second insert, to be placed between the BGH/polyA tail and the insulator, comprised a TUPV-specific 3′ barcode. The third insert, to be placed downstream of the insulator, comprised a BpiI site and a TUPV-specific sticky end. In this manner, 9 TUPVs each with their own unique 5′ and 3′ barcodes and 5′ and 3′ sticky ends were cloned (pPD471–479). This initial library uses a CMV promoter as the core promoter for each TU, which was placed upstream of a multiple cloning site (MCS). A second library of 9 TUPVs was then constructed by replacing the CMV promoter with the CAG promoter by restriction enzyme digest with SnaBI and NheI (pPD561–569). A third library of 9 TUPVs was constructed by replacing the promoter with EF1alpha (between MluI and NheI) and the MCS replaced with an EBFP2-P2A-BlastR gene (between NheI and NotI) (pJM450–458). Although this third library no longer contains the full pcDNA MCS, it retains the NheI and NotI genes that flank the COMET ZFa and ZFi and many of the RaZFa components.

### Transferring COMET parts into mMoClo

COMET reporters and ZFa were transferred into TUPVs using restriction enzyme cloning. To construct mKate2 reporters in TUPV1, mKate2 was cloned into the MCS of pPD561 using NheI and NotI restriction sites downstream of a CAG promoter to create pHIE041. Binding site arrays were PCR-amplified from pPD152, pPD287, pPD290, pPD296, pPD063, pPD069, and pPD095 and inserted to replace CAG in pHIE041 using BglII and NheI, resulting in pHIE042–049. To construct constitutively expressed VP16-ZF1a in TUPV2 (pJM466), the EBFP-P2A-BlastR in pJM451 was replaced with PCR-amplified VP16-ZF1a from pPD100 using NheI and NotI.

### mMoClo assembly of integration vectors

The mMoClo integration vectors were assembled through a BpiI-mediated Golden Gate reaction. Each 20 μL reaction comprised 2 µL 10× T4 ligase buffer, 2 µL 10× BSA (1 mg/mL stock), 0.8 µL BpiI-FD, 0.8 µL T4 DNA Ligase (400 U/µL stock), 20 fmol integration vector backbone (pPD630), and 40 fmol of each transcription unit and linker plasmid to be inserted. The reaction was incubated at 37 °C for 15 min, then subjected to 55 iterations of thermocycling (37 °C for 5 min, 16 °C for 3 min, repeat), followed by 37 °C for 15 min, 50 °C for 5 min, 80 °C for 10 min to terminate the reactions; then the mixture was cooled to room temperature (optionally held at 4 °C if the reaction ran overnight) and placed on ice prior to immediate transformation into bacteria.

### Plasmid preparation

TOP10 *E. coli* were grown overnight in 100 mL of LB with the appropriate selective antibiotic. The following morning, cells were pelleted at 3000*g* for 10 min and then resuspended in 4 mL of a solution of 25 mM Tris pH 8.0, 10 mM EDTA, and 15% sucrose. Cells were lysed for 15 min by addition of 8 mL of a solution of 0.2 M NaOH and 1% SDS, followed by neutralization with 5 mL of 3 M sodium acetate (pH 5.2). Precipitate was pelleted by centrifugation at 9000*g* for 20 min. Supernatant was decanted and treated with 3 µL of RNAse A (Thermo Fisher) for 1 h at 37 °C. 5 mL of phenol chloroform was added, and the solution was mixed and then centrifuged at 7500*g* for 20 min. The aqueous layer was removed and subjected to another round of phenol chloroform extraction with 7 mL of phenol chloroform. The aqueous layer was then subjected to an isopropanol precipitation (41% final volume isopropanol, 10 min at room temperature, 9000*g* for 20 min), and the pellet was briefly dried and resuspended in 420 µL of water. The DNA mixture was incubated on ice for at least 12 h in a solution of 6.5% PEG 20,000 and 0.4 M NaCl (1 mL final volume). DNA was precipitated with centrifugation at maximum speed for 20 min. The pellet was washed once with ethanol, dried for several h at 37 °C, and resuspended for several h in TE buffer (10 mM Tris, 1 mM EDTA, pH 8.0). DNA purity and concentration were confirmed using a Nanodrop 2000 (Thermo Fisher).

### Cell culture

The HEK293FT cell line was purchased from Thermo Fisher/Life Technologies (RRID: CVCL_6911) and was not further authenticated. The HEK293FT-LP cell line was a gift from Ron Weiss and was authenticated by flow cytometric analysis of EYFP expression, which was shown to be homogenous and stable over time—a pattern which is consistent with the original description of this cell line^33^. Cells were cultured in DMEM (Gibco 31600-091) with 10% FBS (Gibco 16140-071), 6 mM L-glutamine (2 mM from Gibco 31600-091 and 4 mM from additional Gibco 25030-081), penicillin (100 U/μL), and streptomycin (100 μg/mL) (Gibco 15140122), in a 37 °C incubator with 5% CO_2_. Cells were subcultured at a 1:5 to 1:10 ratio every 2–3 d using Trypsin-EDTA (Gibco 25300-054). The HEK293FT cell line and the HEK293FT-LP cell line tested negative for mycoplasma with the MycoAlert Mycoplasma Detection Kit (Lonza Cat #LT07-318).

### Transfection experiments

Experiments were conducted by transient transfection of HEK293FT cells using the calcium phosphate method. For transfection experiments, cells were plated at a minimum density of 1.5 × 10^5^ cells/well in a 24-well plate in 0.5 mL of DMEM, supplemented as described above. After at least 6 h, by which time the cells had adhered to the plate, they were transfected via the calcium phosphate method. Plasmids for each experiment were mixed in H_2_O, and 2 M CaCl_2_ was added to a final concentration of 0.3 M CaCl_2_. The exact DNA amounts added to the mix per well and plasmid details for each experiment are listed in the following sections and can be cross-referenced with the tables in Supplementary Data 3 for further details. This mixture was added dropwise to an equal-volume solution of 2× HEPES-Buffered Saline (280 mM NaCl, 0.5 M HEPES, 1.5 mM Na_2_HPO_4_) and gently pipetted up and down four times. After 2.5–4 min, the solution was mixed vigorously by pipetting eight times. 100 µL of this mixture was added dropwise to the plated cells, and the plates were swirled gently. The next morning, the medium was aspirated and replaced with fresh medium. In some assays, fresh medium contained 0.05% DMSO or 0.05% DMSO with 0.1 µM rapamycin. At 36–48 h post-transfection and at least 24 h post-media change, cells were harvested for flow cytometry with FACS Buffer (PBS pH 7.4 with 2–5 mM EDTA and 0.1% BSA) or with Trypsin-EDTA, which was then quenched with medium, and the resulting cell solution was added to at least two volumes of FACS buffer. Cells were spun at 150*g* for 5 min, FACS buffer was decanted, and fresh FACS buffer was added. All experiments were performed in biologic triplicate.

### Western Blotting

For western blotting, HEK293FT cells were plated at 7.5 × 10^5^ cells/well in 2 mL of DMEM and transfected as above, using 400 μL of transfection reagent per well (the reaction scales with the volume of medium). At 36–48 h after transfection, the cells were lysed with 500 μL of RIPA (150 mM NaCl, 50 mM Tris-HCl pH 8.0, 1% Triton X-100, 0.5% sodium deoxycholate, 0.1% sodium dodecyl sulfate) with protease inhibitor cocktail (Pierce/Thermo Fisher cat# A32953) and incubated on ice for 30 min. The lysate was cleared by centrifugation at 14,000*g* for 20 min at 4 °C and the supernatant was harvested. A BCA assay was performed to determine protein concentration, and after a 10-min incubation with Lamelli buffer (final concentration 60 mM Tris-HCl pH 6.8, 10% glycerol, 2% sodium dodecyl sulfate, 100 mM dithiothreitol, and 0.01% bromophenol blue) at 70 °C, 0.5 μg of total protein was loaded onto a 4-15% Mini-PROTEAN TGX Precast Protein Gel (Bio-Rad) and run at 50 V for 10 min followed by 100 V for at least 1 h. Wet transfer was performed onto an Immuno-Blot PVDF membrane (Bio-Rad) for 45 min at 100 V. Ponceau-S staining was used to confirm successful transfer. Membranes were blocked for 30 min with 3% milk in Tris-buffered saline pH 8.0 (TBS pH 8.0: 50 mM Tris, 138 mM NaCl, 2.7 mM KCl, HCl to pH 8.0), washed once with TBS pH 8.0 for 5 min, then incubated for 1 h at room temperature or overnight at 4 °C in primary solution antibody (Mouse-anti-FLAG M2 (Sigma F1804, RRID: AB_262044), diluted 1:1000 in 3% milk in TBS pH 8.0). Primary antibody solution was decanted, and the membrane was washed once with TBS pH 8.0 then twice with TBS pH 8.0 with 0.05% Tween, for 5 min each. Secondary antibody (HRP-anti-Mouse (CST 7076, RRID: AB_330924), diluted 1:3000 in 5% milk in TBST pH 7.6 (TBST pH 7.6: 50 mM Tris, 150 mM NaCl, HCl to pH 7.6, 0.1% Tween)) was applied for 1 h at room temperature, and the membrane was washed three times for 5 min each time with TBST pH 7.6. The membrane was incubated with Clarity Western ECL Substrate (Bio-Rad) for 5 min, and then exposed to film, which was developed and scanned. Images were cropped with Photoshop CC (Adobe). No other image processing was employed. Original images are provided in Source Data.

The western blot shown in Supplementary Fig. 12f was conducted twice with comparable results. The first experiment included only the RaZFa component (no additional loading control) to confirm the presence of only one band in each lane (data not shown). In the second experiment, 40 ng of pPD798 (encoding a 3x-FLAG tagged NanoLuciferase) was co-transfected with the RaZFa components to provide a control for loading and transfection.

### Analytical flow cytometry

Flow cytometry was run on a BD LSRII or BD LSR Fortessa Special Order Research Product (Robert H. Lurie Cancer Center Flow Cytometry Core). The lasers and filter sets used for data acquisition are listed in Supplementary Table 3. Approximately 2000–3000 single, transfected cells were analyzed per sample.

### Flow Cytometry Data Analysis

Samples were analyzed using the FlowJo v10 software (FlowJo, LLC). Fluorescence data were compensated for spectral bleed-through. As illustrated in Supplementary Fig. 15, the HEK293FT cell population was identified by FSC-A vs. SSC-A gating, and singlets were identified by FSC-A vs. FSC-H gating. To distinguish transfected and non-transfected cells, a control sample of cells was generated by transfecting cells with a mass of pcDNA (empty vector) equivalent to the mass of DNA used in other samples in the experiment. For the single-cell subpopulation of the pcDNA-only sample, a gate was made to identify cells that were positive for the constitutively driven fluorescent protein used as a transfection control in other samples, such that the gate included no more than 1% of the non-fluorescent cells. The mean fluorescence intensity (MFI) of the single-cell transfected population was calculated and exported for further analysis.

To calculate reporter expression, MFI in the FITC channel was averaged across three biologic replicates. From this number, the autofluorescence of the cells was subtracted. To calculate the autofluorescence of the cells, in each experiment, a control group of cells transfected with DNA encoding the fluorescent protein transfection control and pcDNA were used. The background-subtracted MFI was converted to Molecules of Equivalent Fluorescein (MEFLs) by multiplying by a coefficient determined in each experiment, as described below. Standard error was propagated through all calculations.

### Conversion of arbitrary units to standardized fluorescence units

As shown in Supplementary Fig. 16, to determine the conversion factor for MFI to MEFLs, Rainbow Calibration Particles (Spherotech, RCP-30-5) or UltraRainbow Calibration Particles (Spherotech URCP-100-2H) were run with each flow cytometry experiment. This reagent contains six (RCP) or nine (URCP) subpopulations of beads, each of a specific size and with a known number of various fluorophores. The total bead population was identified by SSC vs. FSC gating, and the subpopulations were identified through two fluorescent channels. The MEFL values corresponding to each subpopulation were supplied by the manufacturer. A calibration curve was generated for the experimentally determined MFI vs. manufacturer-supplied MEFLs, and a linear regression was performed with the constraint that 0 MFI equals 0 MEFLs. The slope from the regression was used as the conversion factor, and error was propagated.

### Integration of cargo into landing pad cell lines

From exponentially growing HEK293LP cells, 0.5 × 10^5^ cells were plated per well (0.5 mL medium) in 24-well format, and cells were cultured for 24 h to allow cells to attach and spread. When cells reached 50–75% confluence, Bxb1 recombinase was co-transfected with the integration vector by lipofection with Lipofectamine LTX with PLUS Reagent (ThermoFisher 15338100). 300 ng of BxB1 expression vector was mixed with 300 ng of integration vector and 0.5 μL of PLUS reagent in a 25 μL total volume reaction, with the remainder of the volume being OptiMEM (ThermoFisher/Gibco 31985062). In a separate tube, 1.9 μL of LTX reagent was mixed with 23.1 μL of OptiMEM. The DNA/PLUS Reagent mix was added to the LTX mix. pipetted up and down four times, and then incubated at room temperature for 5 min. 50 μL of this transfection mix was added dropwise to each well of cells, which was mixed by gentle swirling. Cells were cultured until the well was ready to split (typically 3 d), without any media changes.

### Selection and expansion of landing pad cell lines

Cells were harvested from the 24-well plate when confluent by trypsinizing and transferring to a single well of a 6-well plate in 2 mL of medium, and then cells were cultured until they reached 50–70% confluence. Then, medium was aspirated and replaced with 2 mL of fresh media containing appropriate selection antibiotic 1 μg/mL puromycin (Invivogen ant-pr) or 6 μg/mL blasticidin (Alfa Aesar/ThermoFisher J61883). Medium was replaced daily with fresh medium containing antibiotics until cell death was no longer evident. Selection was first performed in puromycin for 7 d, then cells were expanded for 7 d without antibiotics. Cells were then cultured in both puromycin and blasticidin to maintain selective pressure until flow sorting.

### Sorting of landing pad cell lines

Cells were harvested by trypsinizing, resuspended at approximately 10^7^ cells per mL in pre-sort medium (DMEM with 10% FBS, 25 mM HEPES (Sigma H3375), and 100 µg/mL gentamycin (Amresco 0304)), and held on ice until sorting was performed. Cells were sorted using a BD FACS Aria 4-laser Special Order Research Product (Robert H. Lurie Cancer Center Flow Cytometry Core) with the optical configuration listed in Supplementary Table 4.

The sorting strategy was as follows: single cells were first gated to exclude all EYFP positive cells (as EYFP positive cells still have an intact landing pad locus, suggesting a mis-integration event occurred) and to include only EBFP2+ cells. Then a gate was drawn on EBFP2 expression, utilizing the line that demonstrated the least amount of silencing (ZF1x12-C_mKate2 + ZF1a) to capture the 90th to 98th percentile of EBFP2-expressing cells (the top 2% were excluded to exclude cells suspected to possess two or more integrated copies of the cargo vector). The gate drawn using this line was used for all other lines as well. No gating was performed on mKate2 reporter expression. 15,000 cells were collected for each line in post-sort medium (DMEM with 20% FBS, 25 mM HEPES, and 100 μg/mL gentamycin), and cells were held on ice until they could be centrifuged at 150*g* for 5 min and resuspended in DMEM. Cells were plated in a 24-well plate and expanded until used in experiments. Gentamycin was included in the culture medium for one week after sorting.

### Experiments involving landing pad cell lines

Stable cell lines were plated in 0.5 mL of DMEM in triplicate in 24-well format at a density expected to generate 50% confluent wells. The day after plating (24 h), cells were harvested with Trypsin-EDTA, as described in *Transfection experiments*. For transfection experiments designed to accompany landing pad line experiments (Fig. 6a, b), cells were plated and transfected 2 d prior to the assay and harvested as described in *Transfection experiments*. Flow cytometry was run on a BD LSR Fortessa, as described in *Analytical flow cytometry*.

For characterization, approximately 10,000 single, EBFP2-expressing cells were analyzed per sample, where EBFP2 is a marker for locus activity in the stable cells and a transfection control for transfected cells. Stable cells were analyzed using higher laser voltages than those used for transfected cells to effectively capture the range of reporter expression conferred by the panel of COMET promoters; thus, the results from this experiment are displayed in separate panels of Fig. 6 even though data collection occurred on the same day.

### COMET model development and analysis

This section provides an integrated discussion of model development, calibration, and analysis to supplement the discussion in the main text. We first describe the development of the core model and then discuss elaborations and models used for comparison. In developing the core model to investigate and predict COMET behavior, we account for two phenomena: cell heterogeneity using a statistical model, and gene regulation using a dynamical model.

### Statistical model

Heterogeneity is represented by simulating genetic programs in a way that resembles their outcomes in cells, which vary in the expression of the components. The in silico population (**Z**) is an *N* × *P* matrix, where *N* is the number of cells (*n* = 1:*N*, for *N* = 200) and *P* is the number of plasmids (*p* = 1:*P*). Components that are encoded on separate plasmids are assigned separate columns. For example, the ZF1a gene is assigned one column and the reporter gene is assigned another column.

**Z** is generated using the constrained sampling method^27^ using the following steps. First, specify parameters for the target marginal distribution of gene expression. Based on flow cytometry measurements of constitutively expressed fluorescent proteins from co-transfected plasmids, the characteristic distribution for each protein was log-bimodal Gaussian;^27^ distributions can be described by the parameters *μ*_1_ = 1.95, *σ*_1_ = 0.3, *μ*_2_ = 3.4, and *σ*_2_ = 0.6 arbitrary units on a log_10_-scaled axis. Second, specify a target correlation coefficient to model gene expression from co-transfected plasmids. A Pearson correlation of *r* = 0.8 was used based on observed correlations. Third, based on the target correlation, specify a lower bound and upper bound of acceptable values. Values should be chosen that are close to the target, such as 0.765 and 0.835. Fourth, generate a joint distribution using the parameters for the marginal distribution and the target correlation coefficient. This output is a candidate *N* × *P* matrix for population variation. Distributions can be generated using the multivariate normal random number generator in MATLAB. Fifth, compute the correlation coefficient matrix (*P* x *P*). Sixth, while any non-diagonal entries in the correlation coefficient matrix are outside of the range of acceptable values specified in step 3, repeat steps 4 and 5. Lastly, for the accepted matrix, normalize the values in each column to a mean of one to obtain the population matrix **Z**. It is useful to plot the generated distributions and correlations to confirm resemblance to the target outcomes.

### ZFa-mediated transcription

Gene regulation is represented using a system of ODEs. The example below depicts a constitutively expressed ZFa inducing the expression of a reporter. Transcription of ZFa RNA scales linearly with plasmid dose. Transcription of reporter RNA depends on ZFa protein concentration via a response function *f*, which is described in subsequent sections. RNA degradation, protein translation, and protein degradation are represented as first-order processes. The *k* terms are fixed parameters that are either defined as equal to 1 unit or are based on a previous estimate: *k*_transcription_ = 1 arbitrary transcription unit, *k*_degRNA_ = 2.7 h^−1^ based on a previous study^51^, *k*_translation_ = 1 arbitrary translation unit, *k*_degZFa_ = 0.35 h^−1^ based on another study^52^, and *k*_degReporter_ *=* 0.029 h^–1^ based on another study^53^.

1 $$\frac{{{\rm{d}}{\mathbf{ZFa}}_{{\mathbf{RNA}}}}}{{{\rm{d}}t}} = k_{{\mathrm{transcription}}} \cdot dose_{{\mathrm{ZFa}}} - k_{{\mathrm{degRNA}}} \cdot {\mathbf{ZFa}}_{{\mathbf{RNA}}},$$

2 $$\frac{{{\rm{d}}{\mathbf{ZFa}}_{{\mathbf{Protein}}}}}{{{\rm{d}}t}} = k_{{\mathrm{translation}}} \cdot {\mathbf{ZFa}}_{{\mathbf{RNA}}} - k_{{\mathrm{degZFa}}} \cdot {\mathbf{ZFa}}_{{\mathbf{Protein}}},$$

3 $$\frac{{{\rm{d}}{\mathbf{Reporter}}_{{\mathbf{RNA}}}}}{{{\rm{d}}t}} = k_{{\mathrm{transcription}}} \cdot f\left( {{\mathbf{ZFa}}_{{\mathbf{Protein}}}} \right) - k_{{\mathrm{degRNA}}} \cdot {\mathbf{Reporter}}_{{\mathbf{RNA}}},$$

4 $$\frac{{{\rm{d}}{\mathbf{Reporter}}_{{\mathbf{Protein}}}}}{{{\rm{d}}t}} = k_{{\mathrm{translation}}} \cdot {\mathbf{Reporter}}_{{\mathbf{RNA}}} - k_{{\mathrm{degReporter}}} \cdot {\mathbf{Reporter}}_{{\mathbf{Protein}}}.$$

Although the rate constants for transcription and translation for both the ZFa and reporter are set equal to 1 unit, these processes differ in living cells. As a result, 1 unit of ZFa RNA can correspond to a different number of molecules in a living cell than 1 unit of reporter RNA, and likewise for 1 unit of each protein. However, importantly, 1 unit for a given species (e.g., reporter protein) can be treated as equivalent across simulation conditions (e.g., ZFa plasmid doses), and these are the comparisons of interest in our analysis.

For a ZFa-inducible promoter, the response function *f* is defined as

5 $$f = \frac{{b + m \cdot w \cdot {\mathbf{ZFa}}_{{\mathbf{Protein}}}}}{{1 + w \cdot {\mathbf{ZFa}}_{{\mathbf{Protein}}}}},$$

where *b* is a non-negative value for TF-independent (leaky or background) transcription; *m* is a unitless value for maximum activation (for ZF1a, *m* ≥ 1) that depends on the number and spacing of binding sites and the TF; and *w* is a positive value related to the steepness of the ZFa dose response. The ZFa variable refers to the simulated protein level—this is a function of plasmid dose, but is in distinct units from and is not equivalent to plasmid dose.

The parameter *m* describes the maximum transcription that a specific ZFa can drive at a promoter with a specific number and spacing of binding sites. An *m* value of 1 is defined for ZF1a with a x1 promoter. We found that values for *m* vary with the number of binding sites (*BS*). This relationship can be approximated by sigmoid functions as shown below for ZF1a. The max argument ensures that *m* does not go below 1 and that it increases monotonically with the number of binding sites:

6 $$m_{{\mathrm{spaced}}} = {\rm{max}}\left( {\frac{{8.5}}{{1 + {\rm{e}}^{ - 0.48\left( {BS - 7.6} \right)}}},1} \right),$$

7 $$m_{{\mathrm{compact}}} = {\rm{max}}\left( {\frac{{41}}{{1 + {\rm{e}}^{ - 0.98\left( {BS - 4.6} \right)}}},1} \right).$$

For TFs that follow similar binding site-response behavior, the sigmoids appear vertically stretched or squashed. This effect can be represented by changing the numerator value in the fraction for the *m* function.

### Calibration

The model was implemented with modifications to RNA production terms to incorporate cell heterogeneity:

8 $$\frac{{{\rm{d}}{\mathbf{ZFa}}_{{\mathbf{RNA}}}}}{{{\rm{d}}t}} = k_{{\mathrm{transcription}}} \cdot z_{i,p_{{\mathrm{ZFa}}}} \cdot dose_{{\mathrm{ZFa}}} - k_{{\mathrm{degRNA}}} \cdot {\mathbf{ZFa}}_{{\mathbf{RNA}}},$$

9 $$\frac{{{\rm{d}}{\mathbf{ZFa}}_{{\mathbf{Protein}}}}}{{{\rm{d}}t}} = k_{{\mathrm{translation}}} \cdot {\mathbf{ZFa}}_{{\mathbf{RNA}}} - k_{{\mathrm{degZFa}}} \cdot {\mathbf{ZFa}}_{{\mathbf{Protein}}},$$

10 $$\frac{{{\rm{d}}{\mathbf{Reporter}}_{{\mathbf{RNA}}}}}{{{\rm{d}}t}} = k_{{\mathrm{transcription}}} \cdot z_{i,p_{{\mathrm{Reporter}}}} \cdot f\left( {{\mathbf{ZFa}}_{{\mathbf{Protein}}}} \right) - k_{{\mathrm{degRNA}}} \cdot {\mathbf{Reporter}}_{{\mathbf{RNA}}},$$

11 $$\frac{{{\rm{d}}{\mathbf{Reporter}}_{{\mathbf{Protein}}}}}{{{\rm{d}}t}} = k_{{\mathrm{translation}}} \cdot {\mathbf{Reporter}}_{{\mathbf{RNA}}} - k_{{\mathrm{degReporter}}} \cdot {\mathbf{Reporter}}_{{\mathbf{Protein}}},$$

where *z* denotes the intracellular and intercellular variation, using values for the *i*th cell and *p*th plasmid. The model was run by iterating through each cell in the population (over a 42 h simulated duration corresponding to a typical experimental duration), and the population mean was calculated.

In experiments from which data were used to estimate parameters, a ZF1a dose response (0, 5, 10, 20, 50, 100, 200 ng plasmid) with a ZF1x6-C promoter-driven reporter (200 ng plasmid) was included as a fiducial marker for normalizing experiment-specific MEFLs to model-specific units that would be consistent across simulations. For each new ZFa, parameters can be estimated from dose response data using the following steps. First, data for the new ZFa are normalized to the within-experiment ZF1a series: to arrive at the *m*-equivalent units required for steps 2 and 3 below, divide the MEFL values for the new ZFa series by the mean of the MEFL values for the [5, 10, 20, 50, 100, 200] ng portion of the ZF1a series, and multiply by 22.4 (this value is determined from the ZF1a experiment in which *m* was originally defined). Second, specify *m* for the new ZFa series using the maximum observed (or expected) reporter expression. Third, determine *b* from the data point for ZFa-independent reporter expression. Lastly, fit *w* by minimizing the sum of squares error between experimental data and simulated population means. The experimental series and simulated series should use the same ZFa plasmid doses, and they should be normalized equivalently such as by dividing by the mean reporter expression of the series. For cases of non-monotonic reporter expression, data points above the ZFa dose yielding maximum reporter expression should not be used to fit *w*, as the response function is intended to describe only the data from zero ZFa plasmid dose through the maximum reporter expression.

### Standard models of transcription

Figure 2 compares the COMET model with standard models of transcription that use more parameters^26^. Fractional activation *f* by a TF (*y*) with promoter affinity *w* and Hill cooperativity *n* for TF-DNA binding, at a promoter that has one binding site, exhibits leaky transcription *α*_0_, and can be maximally activated by the TF to an amount *α*, is represented as

12 $$f = \frac{{a_0 + a\left( {w{\mathbf{y}}} \right)^n}}{{1 + \left( {w{\mathbf{y}}} \right)^n}}.$$

This formulation can be extended to other scenarios. For two TFs (*y*_1_ and *y*_2_) with respective maximal activation *α*_1_ and *α*_2_, a combined activation *α*_12_, and TF cooperativity *ρ* for RNAP recruitment, at a promoter with one site per TF, the formulation is

13 $$f = \frac{{a_0 + a_1\left( {w_1{\mathbf{y}}_1} \right)^{n_1} \, + \, a_2\left( {w_2{\mathbf{y}}_2} \right)^{n_2} \, + \, a_{12}\rho \left( {w_1{\mathbf{y}}_1} \right)^{n_1}\left( {w_2{\mathbf{y}}_2} \right)^{n_2}}}{{1 \, + \, \left( {w_1{\mathbf{y}}_1} \right)^{n_1} \, + \, \left( {w_2{\mathbf{y}}_2} \right)^{n_2} \, + \, \rho \left( {w_1{\mathbf{y}}_1} \right)^{n_1}\left( {w_2{\mathbf{y}}_2} \right)^{n_2}}}.$$

If in this scenario both TFs are the same (one TF species can bind up to two sites), and additionally if maximal activation is 100% (*α* = 1), this simplifies to

14 $$f = \frac{{a_0 + 2\left( {w{\mathbf{y}}} \right)^n \, + \, \rho \left( {w{\mathbf{y}}} \right)^{2n}}}{{1 + 2\left( {w{\mathbf{y}}} \right)^n \, + \, \rho \left( {w{\mathbf{y}}} \right)^{2n}}}.$$

In a scenario without Hill cooperativity for TF-DNA binding (*n* = 1) and without TF cooperativity (*ρ* = 1), this further simplifies to

15 $$f = \frac{{a_0 + 2w{\mathbf{y}} + \left( {w{\mathbf{y}}} \right)^2}}{{1 + 2w{\mathbf{y}} + \left( {w{\mathbf{y}}} \right)^2}}.$$

We extend the above case to any number of binding sites. Adding sites could affect *ρ* for each term in the numerator and denominator, but for simplicity we constrain the possible values by assuming all *ρ* = 1. This assumption is applied in the lower plots of the first and second landscapes in Fig. 2c. Examples are shown below for three, four, five, and six binding sites. Coefficients are derived using Pascal’s triangle:

16 $$f_3 = \frac{{a_0 + 3\left( {w{\mathbf{y}}} \right)^n \, + \, 3\left( {w{\mathbf{y}}} \right)^{2n} \, + \, \left( {w{\mathbf{y}}} \right)^{3n}}}{{1 + 3\left( {w{\mathbf{y}}} \right)^n \, + \, 3\left( {w{\mathbf{y}}} \right)^{2n} \, + \, \left( {w{\mathbf{y}}} \right)^{3n}}},$$

17 $$f_4 = \frac{{a_0 + 4\left( {w{\mathbf{y}}} \right)^n \, + \, 6\left( {w{\mathbf{y}}} \right)^{2n} \, + \, 4\left( {w{\mathbf{y}}} \right)^{3n} \, + \, \left( {w{\mathbf{y}}} \right)^{4n}}}{{1 + 4\left( {w{\mathbf{y}}} \right)^n \, + \, 6\left( {w{\mathbf{y}}} \right)^{2n} \, + \, 4\left( {w{\mathbf{y}}} \right)^{3n} \, + \, \left( {w{\mathbf{y}}} \right)^{4n}}},$$

18 $$f_5 = \frac{{a_0 + 5\left( {w{\mathbf{y}}} \right)^n \, + \, 10\left( {w{\mathbf{y}}} \right)^{2n} \, + \, 10\left( {w{\mathbf{y}}} \right)^{3n} \, + \, 5\left( {w{\mathbf{y}}} \right)^{4n} \, + \, \left( {w{\mathbf{y}}} \right)^{5n}}}{{1 + 5\left( {w{\mathbf{y}}} \right)^n \, + \, 10\left( {w{\mathbf{y}}} \right)^{2n} \, + \, 10\left( {w{\mathbf{y}}} \right)^{3n} \, + \, 5\left( {w{\mathbf{y}}} \right)^{4n} \, + \, \left( {w{\mathbf{y}}} \right)^{5n}}},$$

19 $$f_6 = \frac{{a_0 + 6\left( {w{\mathbf{y}}} \right)^n \, + \, 15\left( {w{\mathbf{y}}} \right)^{2n} \, + \, 20\left( {w{\mathbf{y}}} \right)^{3n} \, + \, 15\left( {w{\mathbf{y}}} \right)^{4n} \, + \, 6\left( {w{\mathbf{y}}} \right)^{5n} \, + \, \left( {w{\mathbf{y}}} \right)^{6n}}}{{1 + 6\left( {w{\mathbf{y}}} \right)^n \, + \, 15\left( {w{\mathbf{y}}} \right)^{2n} \, + \, 20\left( {w{\mathbf{y}}} \right)^{3n} \, + \, 15\left( {w{\mathbf{y}}} \right)^{4n} \, + \, 6\left( {w{\mathbf{y}}} \right)^{5n} \, + \, \left( {w{\mathbf{y}}} \right)^{6n}}}.$$

For the third and fourth landscapes in Fig. 2c, *m* values for spaced and compact promoters were substituted for *α* in each term of the numerator and denominator. As an example, the equation for three sites is

20 $$f_3 = \frac{{a_0 + 3m_1\left( {w{\mathbf{y}}} \right)^n \, + \, 3m_2\left( {w{\mathbf{y}}} \right)^{2n} \, + \, m_3\left( {w{\mathbf{y}}} \right)^{3n}}}{{1 + 3m_1\left( {w{\mathbf{y}}} \right)^n \, + \, 3m_2\left( {w{\mathbf{y}}} \right)^{2n} \, + \, m_3\left( {w{\mathbf{y}}} \right)^{3n}}}.$$

Since *m* values can exceed 1, *f* no longer represents fractional activation defined with the range of zero to one. This interpretational note also applies to *f* in the COMET model.

To investigate modes of transcriptional regulation independent of the effects of cell heterogeneity, the plots in Fig. 2c, d depict homogeneous (one-cell) expression (whereas the fits shown as lines in Fig. 2a depict heterogeneous population means). To compare the most salient features of each landscape in Fig. 2c, simulations were conducted using the parameter values in Supplementary Table 5, and outcomes were scaled for a maximum attainable value of 1 within each model.

### Mechanistic model of transcription

To assess whether the concise COMET model is consistent with a more detailed representation of gene regulation, we developed a model that more granularly represents interactions between molecular components (Supplementary Fig. 8d–f). The components include those with tunable properties (TF (ZFa), ZFi, and free AD, and for which doses can be specified) and those without tunable properties (RNAP and single-site reporter DNA). The RNAP variable broadly represents the ensemble of factors that are recruited to initiate transcription, and this variable can bind TF or AD. For simplicity, the promoter for the reporter has one site that can be either unoccupied or occupied by TF or ZFi, and there is no TF-independent transcription.

ODEs were run to steady state and used the initial conditions and parameters below. Here, the goal was not to estimate parameter values, and therefore the values are based not on specific intracellular concentrations or rate constants, but rather on those that we observed to produce steady-state trends that were interpretable and resembled the experimentally measured dose responses.

Initial values for the variables are as follows. Reporter DNA: 10 units. RNAP: 200 units. TF: dose response ranging from 0 to 200 units. ZFi: 0 units; 200 if present at a constant amount; or a dose response of [5, 10, 20, 50, 100, 200]. AD: 0 units; 200 if present at a constant amount; or a dose response of [5, 10, 20, 50, 100, 200].

Parameters for the reactions (in distinct arbitrary units) are as follows. Association of TF (or ZFi) and DNA: *k*_a_ = 1 a.u. Dissociation of TF (or ZFi) and DNA: *k*_d_ = 100 a.u.; or varied as [500, 200, 100, 50, 20]. Association of TF (or AD) and RNAP: *k*_f_ = 1 a.u. Dissociation of TF (or AD) and RNAP: *k*_r_ = 20 a.u.; or varied as [100, 50, 20, 10, 5].

Relevant metrics are *k*_a_/*k*_d_ for the TF-DNA and ZFi-DNA interactions and *k*_f_/*k*_r_ for RNAP recruitment by TF or AD. The DNA.TF.RNAP variable was used as a proxy for reporter readout. We made the following observations. First, the simulations qualitatively resembled experimental dose response trends. Second, increasing *k*_f_, *k*_a_, or TF dose led to more DNA.TF.RNAP (within a typical TF dose range). At values far above this range, or with excess non-productive components such as free AD, the dose response became non-monotonic due to non-productive sequestration of components. Third, the effect of ZFi was to decrease DNA.TF.RNAP by occupying the reporter promoter non-productively. Lastly, depending on the initial values of the components, TF dose responses differed in two key ways—the maximum value for DNA.TF.RNAP and the steepness of the dose response; importantly, these features are captured by *m* and *w* respectively in the COMET model.

### Transcriptional inhibition

The model used to generate predictions presented in Fig. 5d, e was developed as follows. Within the COMET framework, a competitive inhibitor is represented as

21 $$f = \frac{{b + m \cdot w_{\mathrm{A}} \cdot {\mathbf{ZFa}}_{{\mathbf{Protein}}}}}{{1 + w_{\mathrm{A}} \cdot {\mathbf{ZFa}}_{{\mathbf{Protein}}} + w_{\mathrm{I}} \cdot {\mathbf{ZFi}}_{{\mathbf{Protein}}}}},$$

where *m* and *w*_A_ correspond to the ZFa, and *w*_I_ corresponds to the inhibitor. However, the observed effect of the inhibitors (Fig. 5) was greater than that predicted by competitive inhibition alone. We found that outcomes with ZFi-DsRed or with a spaced promoter could be explained by also accounting for a decrease in effective cooperativity at the promoter. Removal of cooperativity from a multi-site promoter is a complex process involving an ensemble of promoter states within cells. For simplicity, we represent this as a non-mechanistic heuristic function that depends upon the amounts and properties of both the ZFa and the ZFi. The value *m* is replaced by a ramp down function from baseline cooperativity without inhibitor to no cooperativity at a high amount of inhibitor:

22 $$f = \frac{b + {\mathrm{max}}\left(\min \left( {\frac{{\left( {\frac{{w_{\mathrm{I}} \cdot {\mathbf{ZFi}}_{{\mathbf{Protein}}}}}{{w_{\mathrm{A}} \cdot {\mathbf{ZFa}}_{{\mathbf{Protein}}}}} \, - \, l} \right)\left( {1 \, - \, m} \right)}}{{u \, - \, l}} + m,m} \right),1\right) \cdot w_{\mathrm{A}} \cdot {\mathbf{ZFa}}_{{\mathbf{Protein}}}}{{1 + w_{\mathrm{A}} \cdot {\mathbf{ZFa}}_{{\mathbf{Protein}}} + w_{\mathrm{I}} \cdot {\mathbf{ZFi}}_{{\mathbf{Protein}}}}},$$

where *l* and *u* are empirically determined values for the weight-normalized ratio of inhibitor to activator at which the ramp down from *m* to 1 begins and ends, respectively.

We found that compared to ZFi, ZFi-DsRed was a more potent inhibitor. Multiplying its weight in the equation by a factor of four improved the fit to data, and ramp down parameters were adjusted accordingly to maintain the shape profile:

23 $$f = \frac{{b + {\mathrm{max}}\left(\min \left( {\frac{{\left( {\frac{{4w_{\mathrm{I}} \cdot {\mathbf{ZFiDsRed}}_{{\mathbf{Protein}}}}}{{w_{\mathrm{A}} \cdot {\mathbf{ZFa}}_{{\mathbf{Protein}}}}} \, - \, 4l} \right)\left( {1 \, - \, m} \right)}}{{4u \, - \, 4l}} + m,m} \right),1\right) \cdot w_{\mathrm{A}} \cdot {\mathbf{ZFa}}_{{\mathbf{Protein}}}}}{{1 + w_{\mathrm{A}} \cdot {\mathbf{ZFa}}_{{\mathbf{Protein}}} + 4w_{\mathrm{I}} \cdot {\mathbf{ZFiDsRed}}_{{\mathbf{Protein}}}}}.$$

For the inhibitor dose responses in Fig. 5d, cooperativity was more readily removed with ZFi-DsRed than with ZFi, and with a spaced promoter than with a compact one. However, cooperativity was maintained with ZFi and a compact promoter, and this effect held across ZF1i mutants and doses reported in Fig. 5e.

### Transcriptional logic gates

In Fig. 8d, we used the standard model from Fig. 2 to investigate properties of AND gates. For simplicity, leaky transcription (*a*_0_) is set to zero and Hill coefficients (*n*_1_ and *n*_2_) are set to one. Figure 8d and Supplementary Fig. 14c show four variations that differ in whether each TF’s maximal activation (*a*_1_ and *a*_2_) is less than or equal to the maximum activation with both present (*a*_12_ = 1), and synergy (*ρ*) is present or absent:

24 $$f = \frac{{a_1w_1{\mathbf{y}}_1 + a_2w_2{\mathbf{y}}_2 + a_{12}\rho w_1w_2{\mathbf{y}}_1{\mathbf{y}}_2}}{{1 + w_1{\mathbf{y}}_1 + w_2{\mathbf{y}}_2 + \rho w_1w_2{\mathbf{y}}_1{\mathbf{y}}_2}}.$$

TFs were assigned identical properties such that landscapes were symmetric about the dose response diagonal. Simulations used the homogeneous model and the parameter values in Supplementary Table 7.

In Fig. 8d, TF dose responses span 0 to 200 ng of plasmid, and target gene expression is linearly scaled to a maximum attainable value of 1. Comparison between experiments and simulations shows that the hybrid COMET promoter exhibits hybrid cooperative activity: it resembles x3-S with either ZFa individually, and it resembles x6-C if both ZFa are present in sufficient amounts.

To explain this effect, we consider a scenario in which a ZFa induces transcription at a x6-C promoter:

25 $$f = \frac{{m_{6{\mathrm{xCompact}}} \cdot w \cdot {\mathbf{ZFa}}}}{{1 + w \cdot {\mathbf{ZFa}}}}.$$

Hypothetically, if the pool of ZFa protein in a cell could be partitioned into two sub-pools of equal concentration, each with access to a distinct set of three alternating sites on the reporter promoter, then if only one sub-pool were active the promoter activity would decrease to

26 $$f = \frac{{m_{3{\mathrm{xSpaced}}} \cdot w \cdot \frac{1}{2}{\mathbf{ZFa}}}}{{1 + w \cdot \frac{1}{2}{\mathbf{ZFa}}}}.$$

If the sub-pools differed in properties that affected *m* and *w*, then they could be treated as distinct TFs:

27 $$f = \frac{{m_{3{\mathrm{xSpacedZFa}}1} \cdot w_1 \cdot {\mathbf{ZFa}}_1}}{{1 + w_1 \cdot {\mathbf{ZFa}}_1}},$$

28 $$f = \frac{{m_{3{\mathrm{xSpacedZFa}}2} \cdot w_2 \cdot {\mathbf{ZFa}}_2}}{{1 + w_2 \cdot {\mathbf{ZFa}}_2}}.$$

An inhibitor for either ZFa would act specifically on the corresponding binding sites, such that maximal inhibition would require inhibitor species that tile both sets of sites.

In the limit of high doses of both ZFa, the contribution of each individually to the total activation becomes

29 $$f = \frac{{m_{6{\mathrm{xCompactZFa}}1{\mathrm{ZFa}}2} \cdot w_1 \cdot {\mathbf{ZFa}}_1}}{{1 + w_1 \cdot {\mathbf{ZFa}}_1 + w_2 \cdot {\mathbf{ZFa}}_2}},$$

30 $$f = \frac{{m_{6{\mathrm{xCompactZFa}}1{\mathrm{ZFa}}2} \cdot w_2 \cdot {\mathbf{ZFa}}_2}}{{1 + w_1 \cdot {\mathbf{ZFa}}_1 + w_2 \cdot {\mathbf{ZFa}}_2}}.$$

Together, these contributions sum to

31 $$f = \frac{{m_{6{\mathrm{xCompactZFa}}1{\mathrm{ZFa}}2} \cdot (w_1 \cdot {\mathbf{ZFa}}_1 + w_2 \cdot {\mathbf{ZFa}}_2)}}{{1 + w_1 \cdot {\mathbf{ZFa}}_1 + w_2 \cdot {\mathbf{ZFa}}_2}}.$$

If both ZFa are identical, this expression becomes identical to the original expression.

Predictions made using the two-input AND gate model also guide the interpretation of Fig. 8e, f. In Fig. 8e, the three-input AND gate uses a similar principle as the two-input AND gate: promoter activity is x2-S with each ZFa individually, and it transitions to x6-C if all three ZFa are present. In Fig. 8f, ZFa and ZFi-DsRed modulate the effective number (x0, x1, x3, x6) and spacing (S = spaced, C = compact) of binding sites, and whether there is competitive inhibition (Y = yes, N = no, Y/N = yes for some sites and no for others); the experimental outcomes align with the expectations in Supplementary Table 8. Among the 16 combinations of the four TF inputs, only the combination with the two activators and no inhibitors exhibited x6-C behavior. The resulting cooperativity leads to higher reporter expression than other combinations with x3-S or x1 behavior.

### Statistical analysis

Statistical details for each experiment are in the figure legends. Unless otherwise stated, there are three independent biologic replicates for each condition. The data shown reflect the mean across these biologic replicates of the mean fluorescence intensity (MFI) of approximately 2,000–3,000 single, transfected cells. Error bars represent the standard error of the mean (S.E.M.). For main figures with heat maps, data are also shown in the corresponding supplemental figure as a bar graph with the mean and S.E.M.

ANOVA tests were performed using the Data Analysis Toolpak in Microsoft Excel. Tukey’s HSD tests were performed with *α* = 0.05. Pairwise comparisons were made using a one-tailed Welch’s *t*-test, which is a version of Student’s *t* test in which the variance between samples is treated as not necessarily equal. The comparisons involved reporter only vs. reporter + ZFa in Fig. 1, Fig. 3; inhibited vs. uninhibited, or more inhibited vs. less inhibited, in Fig. 5; no binding sites vs. one binding site in Supplementary Fig. 10; DMSO vs. rapamycin in Fig. 7; and summed individual cases vs. co-expression in Fig. 8. For each comparison, the null hypothesis was that two samples were equal, and the alternative was that the latter was greater. The threshold for significance was set at 0.05. To decrease the false discovery rate, the Benjamini–Hochberg (BH) procedure was applied to each set of tests per figure panel; in all tests, after the BH procedure, the null hypothesis was rejected for *p*-values < 0.05. The outcome of each statistical test is indicated in the figure captions.

### Reporting summary

Further information on research design is available in the Nature Research Reporting Summary linked to this article.

## Supplementary information

Supplementary Information

Description of Additional Supplementary Files

Supplementary Data 1

Supplementary Data 2

Supplementary Data 3

Supplementary Software

Reporting Summary

## Data Availability

All reported experimental data are included as Source Data. The raw datasets generated during and/or analyzed during the current study are available from the corresponding author on reasonable request. Plasmid maps for all plasmids reported in this study are provided as annotated GenBank files in Source Data. The majority of the plasmids used in this study are deposited with and distributed by Addgene, including complete and annotated GenBank files, at https://www.addgene.org/Joshua_Leonard/. COMET plasmids with Addgene numbers ranging from #138717 to #138747 are available as individual plasmids. COMET plasmids with Addgene numbers ranging from #138749 to #138940 are available as individual plasmids or together as a 192-plasmid kit, which includes plasmids not characterized in this study. mMoClo plasmids have Addgene numbers ranging from #139212 to #139278 and are available as individual plasmids or together as a 67-plasmid kit, which includes some plasmids not characterized in this study​. The exceptions are plasmids pPD610, pPD611-pPD619, pPD630—these are not deposited with Addgene. Plasmids pPD610 (BxB1 Recombinase Expression Vector), pPD612 (pLink2), pPD614 (pLink4), and pPD618 (pLink8), and pPD630 (Destination Vector) were obtained through a Material Transfer Agreement with the Massachusetts Institute of Technology (MIT) and are available from Ron Weiss at MIT upon reasonable request (Weiss Lab plasmid names are given in parentheses, above). The series pPD611-pPD619 comprise linker vectors for mMoClo that have been superseded by an extended set that is deposited with Addgene, as described above; pPD611, pPD613, pPD615, pPD616, pPD617, and pPD619 are available from the corresponding author on reasonable request. This study uses data obtained from the following Addgene plasmids, as described in more detail in Methods: #63798, #14893, #58855, #58877, #58876, #78099, #74285, #61425.

## Source data

Source Data

## Code availability

MATLAB code for COMET can be found at https://github.com/leonardlab/COMET. v1.0.1, which was used in this study, is provided in a ZIP file titled Supplementary Software and on Zenodo^54^. All code is provided under an open source license.
