# Supplementary material for: The COMET toolkit for composing customizable genetic programs in mammalian cells
Source: Nat Commun. 2020 Feb 7;11:779. doi: 10.1038/s41467-019-14147-5 (PMC7005830; doi:10.1038/s41467-019-14147-5)
Supplement: Supplementary file 2 — Description of Additional Supplementary Files [file 41467_2019_14147_MOESM2_ESM.pdf]

Title: Supplementary Data 1.
Description: List of plasmids used.

Title: Supplementary Data 2.
Description: Features of mMoClo plasmids.

Title: Supplementary Data 3.
Description: Doses of plasmids used in all transfection experiments.
