# Supplementary material for: The COMET toolkit for composing customizable genetic programs in mammalian cells
Source: Nat Commun. 2020 Feb 7;11:779. doi: 10.1038/s41467-019-14147-5 (PMC7005830; doi:10.1038/s41467-019-14147-5)
Supplement: Supplementary file 6 — Supplementary Software [file 41467_2019_14147_MOESM6_ESM.zip › Supplementary Software/README.rtf]

The COMET toolkit for composing customizable genetic programs in mammalian cellsDOI 10.5281/zenodo.3533966Description of files: generate_TXF_distribution.m: File for generating a matrix representing a heterogeneous population of cells transfected withone or more plasmids (based upon the cell harvest method used in this study).Z_TXF.mat: An output from generate_TXF_distribution.m for 200 cells transfected with up to six plasmids.model_ZFa.m: Simulation of ZFa-inducible gene expression.model_ZFa_ZFi_competitive.m: Simulation of ZFa-inducible and ZFi-inhibitable gene expression. The ZFi effect is representedas purely competitive without affecting cooperative RNAPII recruitment.model_ZFa_ZFi_dual.m: Simulation of ZFa-inducible and ZFi-inhibitable gene expression. The ZFi effect is represented as bothcompetitive and affecting cooperative RNAPII recruitment.model_ZFa_ZFiDsRed_dual.m: Simulation of ZFa-inducible and ZFi-DsRed-inhibitable gene expression. The ZFi-DsRed effect isrepresented as both competitive and affecting cooperative RNAPII recruitment.1. System requirementsThe code can be run using Matlab (https://www.mathworks.com/products/matlab.html) and run on an operating system thatsupports Matlab. Code was developed and tested on macOS Sierra.2. Installation guideNo other specific installations are required.3. DemoFiles can be run in the Matlab command line with the input arguments described and examples provided at the start of eachfile, and they will produce the output arguments as described.The expected run time for each function, other than for generate_TXF_distribution.m, is << 1 second on a standard desktopcomputer.4. Instructions for useParameter values for new TFs and promoters can be estimated as described in the Supplementary Information.
