# Supplementary material for: The COMET toolkit for composing customizable genetic programs in mammalian cells
Source: Nat Commun. 2020 Feb 7;11:779. doi: 10.1038/s41467-019-14147-5 (PMC7005830; doi:10.1038/s41467-019-14147-5)
Supplement: Supplementary file 7 — Reporting Summary [file 41467_2019_14147_MOESM7_ESM.pdf]

nature research

Corresponding author(s): Joshua Leonard

Reporting Summary

Nature Research wishes to improve the reproducibility of the work that we publish. This form provides structure for consistency and transparency in reporting. For further information on Nature Research policies, see Authors & Referees and the Editorial Policy Checklist.

Statistical parameters

When statistical analyses are reported, confirm that the following items are present in the relevant location (e.g. figure legend, table legend, main text, or Methods section).

n/a	Confirmed	
☐	☒	The exact sample size (*n*) for each experimental group/condition, given as a discrete number and unit of measurement
☐	☒	An indication of whether measurements were taken from distinct samples or whether the same sample was measured repeatedly
☐	☒	The statistical test(s) used AND whether they are one- or two-sided<br>*Only common tests should be described solely by name; describe more complex techniques in the Methods section.*
☒	☐	A description of all covariates tested
☐	☒	A description of any assumptions or corrections, such as tests of normality and adjustment for multiple comparisons
☐	☒	A full description of the statistics including central tendency (e.g. means) or other basic estimates (e.g. regression coefficient) AND variation (e.g. standard deviation) or associated estimates of uncertainty (e.g. confidence intervals)
☐	☒	For null hypothesis testing, the test statistic (e.g. *F*, *t*, *r*) with confidence intervals, effect sizes, degrees of freedom and *P* value noted<br>*Give P values as exact values whenever suitable.*
☒	☐	For Bayesian analysis, information on the choice of priors and Markov chain Monte Carlo settings
☒	☐	For hierarchical and complex designs, identification of the appropriate level for tests and full reporting of outcomes
☐	☒	Estimates of effect sizes (e.g. Cohen's *d*, Pearson's *r*), indicating how they were calculated
☐	☒	Clearly defined error bars<br>*State explicitly what error bars represent (e.g. SD, SE, CI)*

Our web collection on statistics for biologists may be useful.

Software and code

Policy information about availability of computer code

Data collection	Flow cytometry data was collected using BD FACS DIVA software.
Data analysis	Flow cytometry data was analyzed using FlowJo software. All custom code for data fitting and modeling was submitted with the manuscript and is additionally available on GitHub. Please see Code and Software Submission Checklist and the Code Availability section of the manuscript for more details.

For manuscripts utilizing custom algorithms or software that are central to the research but not yet described in published literature, software must be made available to editors/reviewers upon request. We strongly encourage code deposition in a community repository (e.g. GitHub). See the Nature Research guidelines for submitting code & software for further information.

Data

Policy information about availability of data

All manuscripts must include a data availability statement. This statement should provide the following information, where applicable:

- Accession codes, unique identifiers, or web links for publicly available datasets
- A list of figures that have associated raw data
- A description of any restrictions on data availability

The datasets generated during and/or analyzed during the current study are available from the corresponding author on reasonable request. Plasmid maps for all

plasmids reported in this study are provided with the paper as GenBank files. The majority of the plasmids used in this study will be deposited on Addgene, with complete and annotated GenBank files on their website. Please see the Data Availability section of the manuscript for more detail. All figures and supplemental figures have associated raw data. There are no restrictions on data availability.

Field-specific reporting

Please select the best fit for your research. If you are not sure, read the appropriate sections before making your selection.

☒ Life sciences ☐ Behavioural & social sciences ☐ Ecological, evolutionary & environmental sciences

For a reference copy of the document with all sections, see nature.com/authors/policies/ReportingSummary-flat.pdf

Life sciences study design

All studies must disclose on these points even when the disclosure is negative.

Sample size	All experiments were conducted in biological triplicate (i.e. N = 3), which is standard in the field.
Data exclusions	In a few experiments, some samples had samples excluded, as the flow cytometer clogged during acquisition of these samples, which were either consumed or destroyed, making repeat acquisition impossible. For experiments in which this happened, further information can be found in Supplementary Tables 11-18.
Replication	For the majority of the experiments in this manuscript, the published data is an experimental repeat of a similar, but not identical, initial pilot experiment. Similar but not identical means that the pilots experiment may have included different controls, comparison samples, or plasmid doses, and the design choices for the published experiments represent iterations on the designs from the pilot experiments. These initial pilot experiments were sometimes performed with mini-prepped plasmid DNA, which is considered less pure than the mutli-step purification method used to generate DNA for all published experiments, which is described in detail in the Methods section. In all cases, the initial pilot and final published experiment gave highly similar, repeatable results. Furthermore, the experiments depicted in Figures 3a and 7d are representative of two identical, independent experiments by two different investigators (PSD and JWD). Additionally, western blotting experiment shown in Figure S12f was performed twice, as described in Methods and Supplementary Information.
Randomization	This is not relevant to the current study, as the same cell line was grown up and then divided between wells prior to transfection.
Blinding	Blinding is not relevant to this study, as flow cytometry data acquisition and analysis is not subject to investigator bias that could be mitigated through blinding.

Reporting for specific materials, systems and methods

Materials & experimental systems

n/a	Involved in the study	
☐	☒	Unique biological materials
☐	☒	Antibodies
☐	☒	Eukaryotic cell lines
☒	☐	Palaeontology
☒	☐	Animals and other organisms
☒	☐	Human research participants

Methods

n/a	Involved in the study	
☒	☐	ChIP-seq
☐	☒	Flow cytometry
☒	☐	MRI-based neuroimaging

Unique biological materials

Policy information about availability of materials

Obtaining unique materials	All plasmids generated in this study will be made freely available on request, and the majority of these plasmids will additionally be deposited on Addgene (www.addgene.org). See Data Availability for more details.

Antibodies

Antibodies used	Monoclonal ANTI-FLAG M2 antibody produced in mouse (Sigma Cat F#1804; RRID: AB_262044)<br>Anti-mouse IgG, HRP-linked Antibody (Cell Signaling Technology Cat# 7076; RRID: AB_330924)
Validation	From Sigma "Our standard antibody validatioIn n processes include verification for each recommended immunodetection

application. Each of the thousands of antibodies in or portfolio are certified through our standard validation process to ensure quality and reproducibility."
From CST "Every CST antibody undergoes rigorous application-specific validation testing customized according to the target and needs of the individual antibody."
In our laboratory, all western blotting experiments include a negative control sample on each membrane. In this manuscript, the negative control is lysate from a sample of cells transfected (at the same time as the other samples in the experiment) with a plasmid constitutively expressing EBFP2 and additional empty, modified pcDNA to ensure that all samples are transfected with the same total mass of plasmid DNA.

Eukaryotic cell lines

Policy information about cell lines

Cell line source(s)	HEK293FT: Life Technologies / Thermo Fisher (Cat# R70007; RRID: CVCL_6911). HEK293FT-LP: Ron Weiss Lab.
Authentication	The HEK293FT line was not authenticated. The HEK293FT-LP line was authenticated only by flow cytometric analysis of EYFP expression, which was shown to be highly homogenous and stable over time, consistent with the original description of this cell line in the literature.
Mycoplasma contamination	These cell lines tested negative for mycoplasma contamination.
Commonly misidentified lines<br>(See ICLAC register)	None.

Flow Cytometry

Plots

Confirm that:

☒ The axis labels state the marker and fluorochrome used (e.g. CD4-FITC).

☒ The axis scales are clearly visible. Include numbers along axes only for bottom left plot of group (a 'group' is an analysis of identical markers).

☐ All plots are contour plots with outliers or pseudocolor plots.

☐ A numerical value for number of cells or percentage (with statistics) is provided.

Methodology

Sample preparation	At 36–48 h post-transfection and at least 24 h post-media change, cells were harvested for flow cytometry with FACS Buffer (PBS pH 7.4 with 2–5 mM EDTA and 0.1% BSA). Cells were spun at 150 x g for 5 min, FACS buffer was decanted, and fresh FACS buffer was added. Alternatively, cells were harvested using a Trypsin-EDTA solution after a single rinse with PBS pH 7.4. After incubation for several minutes at 37 degrees Celsius, the Trypsin was quenched with EDTA and the cell suspension was added to a tube containing FACS buffer, which was then centrifuged as above, and then the cells were resuspended in fresh FACS buffer.
Instrument	Analytical flow cytometry was run on a BD LSRII or BD LSR Fortessa Special Order Research Product (Robert H. Lurie Cancer Center Flow Cytometry Core). FACS was conducted on a BD FACS Aria SORP (Robert H. Lurie Cancer Center Flow Cytometry Core). Instrument configurations can be found in Supplementary Table 19 and 20.
Software	Samples were analyzed using FlowJo v10 software (FlowJo, LLC).
Cell population abundance	Sorted cells were cultured after sorting and then later analyzed for fluorescence expression by flow cytometry. The sorted population was EYFP-/EBFP+. Upon analysis, the 16 sorted lines were on average 85.2% EBFP+ (STDEV 6.6%, Range 76.5% to 97.8%). In a separate experiment, we found that the 16 sorted lines were on average 99.8% EYFP- (STDEV 0.2%, Range 99.1% to 100%).
Gating strategy	The HEK293FT cell population was identified by FSC-A vs. SSC-A gating, and singlets were identified by FSC-A vs. FSC-H gating. To distinguish transfected and non-transfected cells, a sample of cells transfected with a mass of pcDNA equivalent to mass of DNA in other samples in the experiment was used. For the single-cell subpopulation of the pcDNA-only sample, a gate was made to identify cells that were positive for the constitutively driven fluorescent protein used as a transfection control in other samples, such that the gate included no more than 1% of the non-fluorescent cells. This is illustrated in Supplementary Figure 15.

☒ Tick this box to confirm that a figure exemplifying the gating strategy is provided in the Supplementary Information.
